# Effectiveness of High-risk Human Papillomavirus Testing for Cervical Cancer Screening in China: A Multicenter, Open-label, Randomized Clinical Trial

**DOI:** 10.1001/jamaoncol.2020.6575

**Published:** 2020-12-30

**Authors:** Junji Zhang, Yuqian Zhao, Yi Dai, Le Dang, Li Ma, Chunxia Yang, Yang Li, Linghua Kong, Lihui Wei, Shulan Zhang, Jihong Liu, Minrong Xi, Long Chen, Xianzhi Duan, Qing Xiao, Guzhalinuer Abulizi, Guonan Zhang, Ying Hong, Xiaohong Gao, Qi Zhou, Xing Xie, Li Li, Mayinuer Niyazi, Zhifen Zhang, Jiyu Tuo, Yiling Ding, Manfei Si, Fei Chen, Li Song, Youlin Qiao, Jinghe Lang

**Affiliations:** 1 Peking Union Medical College Hospital, Chinese Academy of Medical Sciences/Peking Union Medical College, Beijing, China; 2 Sichuan Cancer Hospital & Institute, Sichuan Cancer Center, School of Medicine, University of Electronic Science & Technology of China, Chengdu, China; 3 National Cancer Center/National Clinical Research Center for Cancer/Cancer Hospital, Chinese Academy of Medical Sciences and Peking Union Medical College, Beijing, China; 4 Dalian Medical University, Dalian, China; 5 West China School of Public Health, Sichuan University, Chengdu, China; 6 Institute of Medical Information, Chinese Academy of Medical Sciences/Peking Union Medical College, Beijing, China; 7 Peking University People’s Hospital, Beijing, China; 8 China Medical University, Shenyang, China; 9 Sun Yat-sen University Cancer Center, Guangzhou, China; 10 West China Second University Hospital, Sichuan University, Chengdu, China; 11 Qingdao Municipal Hospital, Qingdao, China; 12 Beijing Tongren Hospital, Capital Medical University, Beijing, China; 13 The Eighth Affiliated Hospital, Sun Yat-sen University, Shenzhen, China; 14 Affiliated Tumor Hospital of Xinjiang Medical University, Urumqi, China; 15 Nanjing Drum Tower Hospital, Nanjing University Medical School, Nanjing, China; 16 Chongqing University Cancer Hospital, Chongqing, China; 17 Women’s Hospital School of Medicine Zhejiang University, Hangzhou, China; 18 Tumor Hospital of Guangxi Zhuang Autonomous Region, Guangxi, China; 19 People's Hospital of Xinjiang Uygur Autonomous Region, Urumqi, China; 20 Hangzhou Women’s Hospital, Hangzhou, China; 21 Hubei Cancer Hospital, Wuhan, China; 22 The Second Xiangya Hospital of Central South University, Changsha, China; 23 Department of Women and Child Health, National Health Commission of China, Beijing, China

## Abstract

**Importance:**

Evidence is needed regarding the introduction of high-risk human papillomavirus (hrHPV) testing into China’s national cervical cancer screening program.

**Objective:**

To evaluate hrHPV testing as a new screening modality for the national program.

**Design, Setting, and Participants:**

This population-based, multicenter, open-label, randomized clinical trial took place across 20 primary health care centers in urban and rural areas across China. At least 3000 women aged 35 to 64 years per site were invited to participate, for a total of 60 732 women evaluated.

**Interventions:**

At baseline, women were randomly assigned to cytology, hrHPV testing, or visual inspection with acetic acid and Lugol iodine (VIA/VILI) (rural only). Women who tested positive for hrHPV were randomized into cytology-triage, VIA/VILI-triage (rural only), or direct colposcopy arms. Regarding primary or triaging tests, women with cytological abnormalities or who tested positive with VIA/VILI were referred to colposcopy. After 24 months, combined screening of cytology, hrHPV testing, and VIA/VILI was performed, and all women with positive results were referred to colposcopy.

**Main Outcomes and Measures:**

The primary outcomes were cervical intraepithelial neoplasia grade 2 or worse (CIN2+) and CIN3+ yields. The secondary outcome was colposcopy referral rate.

**Results:**

A total of 60 732 women were included in this study, with median (interquartile range) age of 47 (41-52) years. Among urban women, 8955 were randomized to cytology and 18 176 to hrHPV genotyping; among rural women, 11 136 were randomized to VIA/VILI, 7080 to cytology, and 15 385 to hrHPV testing. Participants who tested positive for hrHPV with direct colposcopy had higher risk ratios for disease yields at baseline (urban hrHPV vs cytology, CIN2+ 2.2 [95% CI, 1.6-3.2] and CIN3+ 2.0 [95% CI, 1.2-3.3]; rural hrHPV vs cytology, 2.6 [95% CI, 1.9-4.0] and 2.7 [95% CI, 2.0-3.6]; rural hrHPV vs VIA/VILI, 2.0 [95% CI, 1.6-2.3] and 2.3 [95% CI, 1.8-3.1]). At 24 months, baseline-negative women in the hrHPV arm had significantly lower risk ratios than those with cytology, or VIA/VILI for CIN2+ (0.3 [95% CI, 0.2-0.5], 0.3 [95% CI, 0.2-0.6]) and CIN3+ (0.3 [95% CI, 0.1-0.6], 0.4 [95% CI, 0.2-0.8]) in rural sites. The colposcopy referral rate for hrHPV-positive rural women was reduced to 2.8% by cytology triage, with significantly higher CIN2+ yields than cytology (2.1 [95% CI, 1.3-2.6]) or VIA/VILI arm (1.6 [95% CI, 1.03-2.1]). Genotyping for hrHPV with cytology triage significantly reduced the colposcopy referral rate compared with cytology (0.8 [95% CI, 0.7-0.9]) for urban women.

**Conclusions and Relevance:**

In this randomized clinical trial, testing for hrHPV was an effective primary screening method in primary health care centers. Incorporating hrHPV testing (polymerase chain reaction–based for urban areas, hybrid capture-based for rural areas) into China’s national screening program is reasonable.

**Trial Registration:**

Chinese Clinical Trial Registry Identifier: ChiCTR1900022530

## Key Points

**Question:**

Does integration of high-risk human papillomavirus (hrHPV) testing into China’s national screening program lead to better outcomes than current primary screening approaches?

**Findings:**

In this randomized clinical trial that included 60 732 women, hrHPV testing as primary screening provided a 2.0-fold to 2.7-fold yield for cervical intraepithelial neoplasia grade 2 or worse and grade 3 or worse compared with cytology or visual inspection with acetic acid and Lugol iodine at baseline. It also resulted in a significantly lower risk of cervical intraepithelial neoplasia grade 2 or worse and grade 3 or worse for baseline-negative women under routine conditions in primary health care settings in rural areas across China.

**Meaning:**

It is reasonable to incorporate hrHPV testing (polymerase chain reaction–based testing for urban areas, hybrid capture–based testing for rural areas) as a primary screening method into China’s current national screening program.

## Teaser

This randomized clinical trial compares the results of high-risk human papillomavirus testing vs current primary screening approaches, assessing screening performance, cost-effectiveness, and service system capacity within China’s national cervical cancer screening program.

## Introduction

Cervical cancer leads to high morbidity and mortality in low-income and middle-income countries.^1^ In 2009, China launched a major initiative to curb cervical cancer,^2^ and the national program was expanded to offer annual screening to 10 million women in 2012. Pap smears and visual inspection with acetic acid and Lugol iodine (VIA/VILI) were recommended as primary screening methods. However, this program covers less than 30% of the actual public health demand.

Testing for high-risk human papillomavirus (hrHPV) has been proven to be effective in screening for cervical cancer.^3,4,5,6^ Large prospective trials outside China have demonstrated the increased effectiveness of HPV-based screening compared with cytology.^7^ Cross-sectional studies in China have found hrHPV testing to be sensitive in the detection of cervical intraepithelial neoplasia grade 2 or worse lesions (CIN2+) but with lower specificity than cytology^8,9^ owing to the high prevalence of hrHPV infection and its transient nature. A cluster randomization study in India reported similar CIN2+ yields by hrHPV testing, cytology, or VIA, although a single round of HPV testing significantly reduced the numbers of advanced cervical cancers.^10,11^

The World Health Organization proposed an intermediate target toward elimination of cervical cancer, namely, the screening of 70% of women aged 35 to 45 years with a high-precision test (with performance similar to the HPV test) by 2030.^12^ This goal places China’s screening system under great pressure because of its large population. The question remains as to whether introducing hrHPV testing into the national program could offer better detection of high-grade CIN cases in routine conditions. This trial was designed to evaluate the screening performance, cost-effectiveness, and service system capacity of hrHPV-based screening when carried out in primary health care settings. We report the clinical findings here, with health economics evaluation and service assessment being reported elsewhere.^13,14,15^

## Methods

### Study Design

This is a multicenter, open-label, randomized clinical trial (trial protocol in Supplement 1).^16^ A total of 2 to 4 sites from each of the 7 included geographical regions were purposively sampled, providing a balance of rural counties (n = 11) and urban districts (n = 10) (eFigure 1 in Supplement 2). The selected sites had been appointed as national cervical cancer screening sites at least 1 year before this study and in the national program within the following few years. One urban site that withdrew from the 24-month screening was excluded. Central ethics approval was obtained from the institutional review board of Peking Union Medical Hospital (No. S-705), and all institutional review boards of the participating hospitals approved this study.

### Participants

Approximately 3000 eligible women aged 35 to 64 years who lived in villages or subdistricts were enrolled from each site, with a total of 60 732 women participating. Free screening was offered regardless of entry into the study. Women were eligible if they had no history of cervical cancer or hysterectomy, had no current pregnancy, could understand the study procedures, and voluntarily participated. All enrolled women provided written informed consent.

### Randomization and Masking

Women were randomized at baseline after providing written informed consent. The random allocation program was generated by statisticians in the Cancer Hospital of Chinese Academy of Medical Sciences and pre-embedded in an ACCESS registration database sent to each site. Participants and staff were not masked to randomization. Urban women were assigned to the hrHPV genotyping or cytology arm at a 2:1 ratio. Women who tested positive for HPV-16/18 were referred to colposcopy, and women who tested positive for other hrHPV subtypes were assigned at a 1:1 ratio to cytology triage or direct colposcopy. Rural women were assigned to the hrHPV, cytology, or VIA/VILI arm at a 1:1:1 ratio. Women who tested positive for hrHPV were assigned at a 1:1:1 ratio to VIA/VILI triage or cytology triage or direct colposcopy. In 4 rural sites where cytology was unavailable, women were assigned to the hrHPV or VIA/VILI arm at a 2:1 ratio; hrHPV-positive women were assigned at a 1:1 ratio to VIA/VILI triage or direct colposcopy.

### Procedures

All procedures were performed by trained local physicians. Standardized materials and equipment for cytology and hrHPV testing were provided. For the baseline screening, women came to the site on an appointed day and local health workers introduced the study procedures. After providing written informed consent, women were interviewed in private rooms to obtain demographic information and then received gynecologic examinations according to the allocation.

For urban women, cervical exfoliate cells were obtained in ThinPrep medium (Hologic Inc) and tested for either cytology or hrHPV. In rural sites, brush specimens (Cervical Sampler, QIAGEN) were obtained for hrHPV testing; cervical specimens were obtained in ThinPrep medium for the cytology arm. For VIA/VILI, gynecologists examined the cervix with the naked eye under a bright halogen focus lamp after applying 5% acetic acid and recorded the result 1 minute later, and Lugol’s iodine was applied if necessary. In urban sites, the polymerase chain reaction-based Cobas 4800 test (Roche Diagnostics) or Liferiver hrHPV genotyping kit (ZJ Bio-Tech) was applied. In rural sites, the hybrid capture-based careHPV (QIAGEN) assay was used.^8^

For cytology and VIA/VILI primary or triage tests, atypical squamous cells with cytology of undetermined significance or worse (ASC-US+) or positive VIA/VILI test results were referred to colposcopy. Under colposcopy, lesion-targeted biopsies were performed. In cases of high-grade cytology abnormalities but negative colposcopy findings, 4-quadrant random biopsy at the squamous column junction and endocervical curettage was performed.

At 24 months, all women, except for those detected with CIN2+ at baseline, were called back. Combined screening of cytology, hrHPV testing, and VIA/VILI (rural only) was performed, and all women with positive test results were referred to colposcopy.

The cervical intraepithelial neoplasia (CIN) and Bethesda systems were used for histology and cytology, respectively. The pathology processing and readings were performed at local hospitals. Cytology was deemed positive if it showed ASC-US+; histology was assessed as positive if CIN or cancer were shown. All positive cytology and histopathology specimens by local pathologists, 10% of random samples of negative cytology, and 20% of negative histopathology samples were reviewed by an external pathologist from the provincial hospital. In China, CIN2 remains the cut-off for treatment. Lesions of CIN2 and CIN3 were treated with loop electrosurgical excision procedure or conization. Invasive cancers were treated with hysterectomy, radiotherapy, and chemotherapy if necessary.

### Outcomes

The primary outcomes were CIN2+ and CIN3+ yields. The secondary outcome was colposcopy referral rates.

### Statistical Analysis

Analyses were performed in the intention-to-screen population. Sample size estimation was based on a comparison of CIN2+ yields at baseline. The CIN2+ yield from the national program was 0.14%,^17^ the pilot study indicated 0.55% for hrHPV testing,^18^ and empirical estimation indicated that the yields from VIA/VILI or cytology might be improved to 0.19%. At the adjusted α of 0.025 after Bonferroni correction, 7055 women per arm at baseline provided 90% power to detect the differences. Assuming 20% loss to follow-up, the sample size was 8819 per arm.

The disease yields, yields ratios, positive rates, colposcopy referral rates, and risk ratios (RRs) were calculated together with 95% CIs by Wilson score method for proportions or by Newcombe-Wilson method for ratios.^19,20^ The denominator was the number of women tested with a valid result at baseline. The hrHPV arms included a second stage of randomization. Inverse proportional weighting was applied to data in each triaging arm to upwardly adjust the sample size of each triaging arm to its size in a primary screening arm and was also used when combining triaging arms to create composite hrHPV arms.^21^ For the adjusted data, 95% CIs were estimated by the bootstrap method (1000 times).^22^

A χ^2^ test was performed to compare proportions. Multiple imputation based on logistic regression was used to account for missing outcomes. For women lost to follow-up, the demographics and baseline results were compared between arms, and no significant differences were found between women who were followed up (eTable 1 in Supplement 2). Reported *P* values were two-sided. A *P* value less than 0.05 or a 95% CI of the ratios below or above 1 were considered statistically significant. Analyses were performed in SAS, version 9.4 (SAS Institute).

## Results

Between May 18, 2015, and September 30, 2016, a total of 60 732 women were randomly allocated and completed the primary screening (eFigure 2a and eFigure 2b in Supplement 2). In urban sites, 8955 women were allocated to cytology and 18 176 to hrHPV testing. In rural sites, 7080 women were allocated to cytology, 11 136 to VIA/VILI, and 15 385 to hrHPV testing. The arms were well balanced for demographic distribution stratified by site (Table 1).

**Table 1.** Baseline Characteristics of Participants for Primary Screening Arms

Characteristic	No. (%)^a^					
	All participants (n = 60 732)	Arm				
		Urban sites		Rural sites		
		hrHPV (n = 18 176)	Cytology (n = 8955)	hrHPV (n = 15 385)	Cytology (n = 7080)	VIA/VILI (n = 11 136)
Age						
Median (IQR)	47 (41-52)	46 (41-51)	46 (42-52)	46 (41-52)	48 (42-53)	47 (41-53)
35-44	24 500 (40.3)	7653 (42.1)	3874 (43.3)	6336 (41.2)	2388 (33.7)	4249 (38.2)
45-54	25 717 (42.3)	7960 (43.8)	3864 (43.1)	6286 (40.9)	3035 (42.9)	4572 (41.0)
55-64	10 515 (17.3)	2563 (14.1)	1217 (13.6)	2763 (18.0)	1657 (23.4)	2315 (20.8)
Marriage status						
Single	143 (0.2)	61 (0.3)	37 (0.4)	21 (0.1)	13 (0.2)	11 (0.1)
Married	57 463 (95.2)	17 186 (94.6)	8470 (94.6)	14 405 (95.5)	6775 (95.7)	10 627 (95.9)
Divorced	1267 (2.1)	563 (3.1)	257 (2.9)	204 (1.4)	106 (1.5)	137 (1.2)
Widowed	1399 (2.3)	352 (1.9)	182 (2.0)	431 (2.9)	155 (2.2)	279 (2.5)
Other	105 (0.2)	14 (0.1)	9 (0.1)	28 (0.2)	31 (0.4)	23 (0.2)
Annual family income (renminbi yuan/y)						
<30 000	27 805 (46.2)	6547 (36.1)	3209 (36.0)	8014 (53.2)	4041 (57.1)	5994 (54.2)
30 000-60 000	21 630 (35.9)	6594 (36.4)	3208 (36.0)	5501 (36.5)	2321 (32.9)	4006 (36.2)
60 000-100 000	6779 (11.3)	3079 (17.0)	1527 (17.1)	999 (6.6)	463 (6.6)	711 (6.4)
>100 000	4007 (6.7)	1899 (10.5)	973 (10.9)	546 (3.6)	236 (3.3)	353 (3.2)
Smoking status						
Current or former smoker	1416 (2.4)	308 (1.7)	211 (2.4)	309 (2.1)	256 (3.6)	332 (3.0)
Never or rarely	58 420 (97.6)	17 619 (98.3)	8669 (97.6)	14 663 (97.9)	6776 (96.4)	10 693 (97.0)
Menopause						
No	31 861 (60.7)	11 330 (62.7)	5668 (63.7)	8661 (59.3)	3868 (55.6)	6202 (57.0)
Peri	3403 (6.5)	1238 (6.9)	633 (7.1)	906 (6.2)	391 (5.6)	626 (5.7)
Post	17 191 (32.8)	5501 (30.4)	2601 (29.2)	5029 (34.5)	2699 (38.8)	4060 (37.3)
Sexual partners in past 3 y						
None	1679 (3.2)	688 (3.8)	289 (3.2)	460 (3.1)	197 (2.8)	320 (2.9)
1	50 498 (96.1)	17 366 (95.5)	8577 (96.2)	14 050 (96.0)	6761 (96.5)	10 505 (96.5)
≥2	358 (0.7)	122 (0.7)	52 (0.6)	123 (0.8)	47 (0.7)	61 (0.6)

Abbreviations: hrHPV, high-risk human papillomavirus; IQR, interquartile range; VIA/VILI, visual inspection with acetic acid and Lugol iodine.

^a^ For missing data, percentages were calculated with available data.

As shown in Table 2, for the urban women in the hrHPV genotyping arm, the positive rate was 9.5% at baseline. Among them, 2.0% tested positive for HPV16/18, 7.3% tested positive for another hrHPV subtype, and 0.3% tested positive for both HPV16/18 and another subtype. The CIN2+ and CIN3+ yields in the hrHPV genotyping arm were 0.7% (n = 122) and 0.3% (n = 61), respectively. For the cytology arm, 5.9% had ASC-US+, 0.4% (n = 36) had CIN2+, and 0.2% (n = 18) had CIN3+. In rural sites, 12.7% of the women in the hrHPV testing arm tested positive, and the CIN2+ and CIN3+ yields were 0.6% (n = 91) and 0.3% (n = 49), respectively; for the cytology arm, 4.2% had ASC-US+, with a CIN2+ yield of 0.4% (n = 25) and a CIN3+ yield of 0.2% (n = 15); for the VIA/VILI arm, 12.3% tested positive, and the CIN2+ and CIN3+ yields were 0.5% (n = 51) and 0.2% (n = 27), respectively. Baseline screening with hrHPV testing detected significantly more CIN2+ than screening with cytology in urban (risk ratio, 1.7 [95% CI, 1.2-2.4]) and rural sites (risk ratio, 1.7 [95% CI, 1.1-2.6]), regardless of the triaging algorithms.

**Table 2.** Population and Outcomes for hrHPV Testing, Cytology, or VIA/VILI Arms at Baseline and at 24-Month Follow-up Screening

Characteristic	Arm							
	Urban sites			Rural sites				
	No. (%) [95% CI]		hrHPV testing vs cytology, RR (95% CI)	No. (%) [95% CI]			RR (95% CI)	
	hrHPV testing (n = 18 176)^a^	Cytology (n = 8955)		hrHPV testing (n = 15 385)^a^	Cytology (n = 7080)	VIA/VILI (n = 11 136)	hrHPV testing vs cytology	hrHPV testing vs VIA/VILI
**Baseline screening**								
Positive	1728 (9.5) [9.1-9.9]	527 (5.9) [5.4-6.4]	1.6 (1.5-1.8)	1949 (12.7) [12.2-13.2]	298 (4.2) [3.8-4.7]	1367 (12.3) [11.7-12.9]	3.0 (2.7-3.4)	1.0 (0.97-1.1)
Colposcopy	1080 (5.9) [5.6-6.3]	414 (4.6) [4.2-5.1]	1.3 (1.2-1.4)	1020 (6.6) [6.2-7.0]	254 (3.6) [3.2-4.0]	1229 (11.0) [10.5-11.6]	1.8 (1.6-2.1)	0.6 (0.6-0.7)
CIN2+ yield	122 (0.7) [0.6-0.8]	36 (0.4) [0.3-0.6]	1.7 (1.2-2.4)	91 (0.6) [0.5-0.7]	25 (0.4) [0.2-0.5]	51 (0.5) [0.3-0.6]	1.7 (1.1-2.6)	1.3 (0.9-1.8)
CIN3+ yield^b^	61 (0.3) [0.3-0.4]	18 (0.2) [0.1-0.3]	1.7 (0.99-2.8)	49 (0.3) [0.2-0.4]	15 (0.2) [0.1-0.3]	27 (0.2) [0.2-0.4]	1.5 (0.8-2.7)	1.3 (0.8-2.1)
**24-mo follow-up screening for baseline test-negative (primary hrHPV testing, cytology, or VIA/VILI) women**								
Participants	12 304 (67.7) [67.0-68.4]	6225 (69.5) [68.6-70.5]	NA	10 048 (65.3) [64.6-66.1]	5349 (75.6) [74.5-76.5]	7368 (66.2) [65.3-67.0]	NA	NA
Positive	1320 (7.3) [6.9-7.7]	739 (8.3) [7.7-8.8]	0.9 (0.8-0.96)	1290 (8.4) [8.0-8.8]	972 (13.7) [13.0-14.6]	1158 (10.4) [9.9-11.0]	0.6 (0.6-0.7)	0.8 (0.7-0.9)
Colposcopy	1142 (6.3) [5.9-6.7]	632 (7.1) [6.6-7.6]	0.9 (0.8-0.98)	1053 (6.8) [6.5-7.3]	904 (12.8) [12.1-13.6]	989 (8.9) [8.4-9.4]	0.5 (0.5-0.6)	0.8 (0.7-0.8)
CIN2+ yield	25 (0.1) [0.1-0.2]	15 (0.2) [0.1-0.3]	0.9 (0.4-1.6)	18 (0.1) [0.1-0.2]	28 (0.4) [0.3-0.6]	39 (0.4) [0.3-0.5]	0.3 (0.2-0.5)	0.3 (0.2-0.6)
CIN3+ yield^b^	5 (0.04) [0.02-0.1]	5 (0.1) [0.06-0.1]	0.5 (0.1-1.8)	9 (0.1) [0.03-0.1]	16 (0.2) [0.1-0.4]	18 (0.2) [0.1-0.3]	0.3 (0.1-0.6)	0.4 (0.2-0.8)

Abbreviations: CIN, cervical intraepithelial neoplasia; hrHPV, high-risk human papillomavirus; NA, not applicable; VIA/VILI, visual inspection with acetic acid and Lugol iodine.

^a^ All women in the hrHPV testing arm had hrHPV testing as primary screening at baseline, regardless of the triage tests.

^b^ At baseline screening, 10 cases of cervical cancer in urban sites, and 6 cases of cervical cancer in rural sites were detected; at the 24-month follow-up screening, 4 cases of cervical cancer in urban sites, and 8 cases of cervical cancer in rural sites were detected.

Among urban women, the RRs of CIN2+ and CIN3+ for baseline primary test-negative women at the 24-month screening were 0.9 (95% CI, 0.4-1.6) and 0.5 (95% CI, 0.1-1.8), respectively. Among rural women, significant reductions in CIN2+ and CIN3+ risk for hrHPV test-negative women were observed compared with cytology (0.3 [95% CI, 0.2-0.5] and 0.3 [95% CI, 0.1-0.6]) and VIA/VILI (0.3 [95% CI, 0.2-0.6] and 0.4 [95% CI, 0.2-0.8]).

The results for different triage strategies are shown in Table 3. At baseline, the CIN2+ and CIN3+ yields were significantly higher for the strategy of direct colposcopy referral with any hrHPV-positive women (adjusted RRs, 2.2 [95% CI, 1.6-3.2] and 2.0 [95% CI, 1.2-3.3]) than for the cytology arm for urban sites. The addition of cytology triage for other hrHPV subtype-positive women improved the CIN2+ and CIN3+ yields compared with the cytology arm but showed no statistical significance (1.3 [95% CI, 0.9-1.9] and 1.5 [95% CI, 0.9-2.6]). In rural sites, significantly higher baseline CIN2+ yields for the hrHPV arm vs cytology arm were found for cytology triage (2.1 [95% CI, 1.3-2.6]) and for hrHPV-positive women with direct colposcopy (2.6 [95% CI, 1.9-4.0]). Significantly higher CIN3+ yields for the hrHPV arm vs cytology arm were also found for hrHPV-positive women with direct colposcopy (2.7 [95% CI, 2.0-3.6]). A similar pattern for disease yields was observed for the hrHPV arm vs VIA/VILI arm for baseline screening.

**Table 3.** Estimated Results Between Primary Screening Arms of Triaging HPV-Positive Women With Cytology, VIA/VILI, or Direct Colposcopy^a^

Characteristic	RR (95% CI)							
	Urban sites		Rural sites					
	hrHPV arms vs cytology arm		hrHPV arms vs cytology arm			hrHPV arms vs VIA /VILI arm		
	Direct colposcopy^b^	Cytology triage^c^	Direct colposcopy^b^	Cytology triage^c^	VIA/VILI triage	Direct colposcopy^b^	Cytology triage^c^	VIA/VILI triage
**Baseline screening**								
Positive	1.6 (1.5-1.8)	0.8 (0.7-0.9)	3.0 (2.7-3.4)	0.7 (0.5-0.8)	0.8 (0.7-0.9)	1.0 (0.97-1.1)	0.2 (0.2-0.3)	0.3 (0.2-0.3)
Colposcopy	2.0 (1.8-2.2)	0.8 (0.8-0.96)	3.5 (3.1-4.0)	0.7 (0.6-0.8)	0.9 (0.8-1.04)	1.1 (1.1-1.2)	0.2 (0.2-0.3)	0.3 (0.3-0.3)
CIN2+ yield	2.2 (1.6-3.2)	1.3 (0.9-1.9)	2.6 (1.9-4.0)	2.1 (1.3-2.6)	1.2 (0.7-1.5)	2.0 (1.6-2.3)	1.6 (1.03-2.1)	0.9 (0.5-1.2)
CIN3+ yield	2.0 (1.2-3.3)	1.5 (0.9-2.6)	2.7 (2.0-3.6)	1.6 (0.8-2.5)	0.6 (0.2-1.04)	2.3 (1.8-3.1)	1.4 (0.7-2.2)	0.5 (0.2-0.9)
**24-mo follow-up screening**								
Positive	0.3 (0.2-0.3)	0.3 (0.3-0.3)	0.2 (0.2-0.3)	0.3 (0.3-0.3)	0.2 (0.2-0.3)	0.3 (0.2-0.3)	0.4 (0.3-0.4)	0.3 (0.3-0.3)
Colposcopy	0.3 (0.2-0.3)	0.3 (0.3-0.3)	0.2 (0.2-0.2)	0.3 (0.3-0.3)	0.2 (0.2-0.2)	0.2 (0.2-0.3)	0.4 (0.4-0.4)	0.3 (0.2-0.3)
CIN2+ yield	0.5 (0.3-0.97)	0.6 (0.4-1.2)	0.6 (0.4-0.93)	0.8 (0.5-1.1)	0.3 (0.2-0.5)	0.8 (0.5-1.1)	0.9 (0.6-1.3)	0.4 (0.2-0.6)
CIN3+ yield	0.2 (0.03-0.8)	0.4 (0.1-1.2)	0.7 (0.4-1.1)	0.2 (0.1-0.5)	0.1 (0.03-0.3)	0.9 (0.5-1.7)	0.3 (0.1-0.7)	0.1 (0.05-0.4)
**24-mo cumulative results**								
Positive	0.8 (0.8-0.9)	0.5 (0.5-0.5)	0.8 (0.8-0.9)	0.4 (0.3-0.4)	0.4 (0.3-0.4)	0.7 (0.6-0.7)	0.3 (0.3-0.3)	0.3 (0.3-0.3)
Colposcopy	0.9 (0.9-1.00)	0.5 (0.5-0.6)	0.9 (0.8-0.9)	0.4 (0.3-0.4)	0.4 (0.3-0.4)	0.7 (0.7-0.7)	0.3 (0.3-0.3)	0.3 (0.3-0.3)
CIN2+ yield	1.6 (1.2-2.2)	1.1 (0.8-1.5)	1.4 (1.1-1.9)	1.3 (0.98-1.7)	0.7 (0.5-0.9)	1.4 (1.1-1.8)	1.3 (1.01-1.7)	0.6 (0.5-0.9)
CIN3+ yield	1.5 (0.97-2.4)	1.2 (0.8-1.9)	1.5 (1.03-2.2)	0.8 (0.5-1.2)	0.3 (0.2-0.5)	1.7 (1.2-2.4)	0.9 (0.7-1.4)	0.4 (0.2-0.6)

Abbreviations: ASC-US+, atypical squamous cells with cytology of undetermined significance or worse; CIN, cervical intraepithelial neoplasia; hrHPV, high-risk human papillomavirus; VIA/VILI, visual inspection with acetic acid and Lugol iodine.

^a^ All data are adjusted for verification bias, not actual numbers. Data are risk ratios (95% CIs). Estimated numbers are presented in eTable 3 in Supplement 2.

^b^ Direct colposcopy encompasses all hrHPV-positive women who were referred to colposcopy directly.

^c^ Cytology triage encompasses women in urban sites who tested HPV-16/18 positive referred to direct colposcopy, other hrHPV subtypes-positive women had cytology triage, cytology ASC-US+ referred to colposcopy; women in rural sites with positive hrHPV had cytology triage, cytology ASC-US+ referred to colposcopy.

At the 24-month screening in urban sites, CIN2+ and CIN3+ yields for hrHPV-positive women with direct colposcopy were significantly lower than those for the cytology arm (CIN2+ 0.5 [95% CI, 0.3-0.97], CIN3+ 0.2 [95% CI, 0.03-0.8]) (Table 3). Genotyping with cytology triage showed lower RRs but no significance compared with the cytology arm. For rural sites, hrHPV-positive women with direct colposcopy had a significantly lower CIN2+ RR than those in the cytology arm (0.6 [95% CI, 0.4-0.93]) at 24 months. Significant reduction in CIN3+ was observed for the cytology triage vs cytology arms (0.2 [95% CI, 0.1-0.5]). Women who tested positive for hrHPV with VIA/VILI triage had significantly lower RRs than the cytology arm for CIN2+ (0.3 [95% CI, 0.2-0.5]) and CIN3+ (0.1 [95% CI, 0.03-0.3]). Compared with those in the VIA/VILI arms, hrHPV-positive women had triage tests showing similar patterns to those reported for the cytology arms.

For the 24-month cumulative results (Table 3), the RR for CIN2+ in hrHPV-positive women with direct colposcopy was significantly higher than that in the cytology arm (1.6 [95% CI, 1.2-2.2]) in urban sites. For rural sites, hrHPV-positive women with direct colposcopy had significantly higher CIN2+ and CIN3+ risks vs those in the cytology arm (1.4 [95% CI, 1.1-1.9] and 1.5 [95% CI, 1.03-2.2]). Significantly lower RRs for CIN2+ and CIN3+ were observed for the VIA/VILI-triage vs cytology arms (0.7 [95% CI, 0.5-0.9] and 0.3 [95% CI, 0.2-0.5]). Compared with those in the VIA/VILI arms, the hrHPV-positive women with triage tests showed similar patterns.

The colposcopy referral rates, if referring all women who tested positive to colposcopy, were significantly higher for hrHPV testing than for the cytology arm in both urban and rural sites (urban: 9.5% vs 5.9%; rural: 12.7% vs 4.2%, *P* < .001) but similar for hrHPV compared with VIA/VILI (12.7% vs 12.3%, *P* = .34) in rural sites. Colposcopy referral rates could be reduced by 70% to 80% if hrHPV-positive women were triaged by cytology (2.8%) or VIA/VILI (3.4%) compared with direct colposcopy in rural sites (eTable 3 in Supplement 2). In urban sites, the strategy of referring women with HPV16/18 to direct colposcopy with cytology triage for other subtypes significantly reduced the number of colposcopies compared with the cytology arm (4.6% vs 5.9%, 0.8 [0.7-0.9]), with no significantly higher disease yields.

In the investigation of the effect of missing outcomes for women lost to follow-up at the 24-month screening, we found a similar pattern to that shown in Table 2 and eTable 2 in the comparison of the arms for primary and secondary outcomes (eTable 4 in Supplement 2).

## Discussion

This trial aimed to assess the effectiveness of introducing hrHPV testing into China’s national program as a primary test compared with cytology and VIA/VILI under the conditions of routine screening in primary health care centers. We found 2.0-fold to 2.7-fold higher disease yields for hrHPV testing with direct colposcopy compared with cytology or VIA/VILI. At 24 months, significantly lower RRs of high-grade CIN for baseline-negative women were observed for hrHPV testing in rural sites. The trial provides further evidence for hrHPV-based screening in a large-scale screening program in China.

Cervical cancer mortality can be reduced by up to 80% with high-quality screening by Papanicolaou tests.^23^ However, false-negative results of Papanicolaou tests accounted for 30% of failures to prevent invasive cancer.^24^ A randomized trial reported by Ronco et al^25^ showed 2-fold higher CIN2+ and CIN3+ yields for hrHPV testing as primary screening than for conventional cytology. In our data, the ratio for hrHPV-positive women with direct colposcopy vs cytology similarly ranged from 2.0 to 2.7 for CIN2+ and CIN3+. A systematic review for hrHPV screening that examined 8 randomized trials, 5 cohort studies, and 1 meta-analysis concluded that although the studies used different protocols, hrHPV-based screening consistently detected more CIN2+ at first-round screening than cytology.^26^

As a simple and low-cost test, VIA/VILI has been widely used in rural China, as well as in other low-income and middle-income countries. Catarino et al^27^ pooled 23 studies focused on visual inspection and estimated that the pooled sensitivity and specificity were 90% and 83%, respectively, which were much higher than those reported in China.^28,29,30^ Women who had negative VIA/VILI results needed to be screened frequently.^31^ The present study’s data show that the RRs of CIN2+ and CIN3+ were significantly lower for hrHPV-negative women compared with VIA/VILI-negative women (0.3 [95% CI, 0.2-0.6] and 0.4 [95% CI, 0.2-0.8]) within 24 months.

Considering that it is not feasible to refer all hrHPV-positive women to colposcopy, different triage strategies for hrHPV-positive women have been assessed. Cox et al^32^ reported that genotyping with a reflex cytology reduced colposcopies by 58% compared with hrHPV testing alone, and this method ranked third among 10 screening strategies in terms of sensitivity. Colposcopy referrals could be reduced by triaging tests but can lead to missed cases.^32,33,34^ However, it is anticipated that the quality of cytology could be improved by sharply reducing the workload from primary screening. Studies have reported a sharp reduction in sensitivity for hrHPV-positive women triaged by visual inspection in both China and Africa.^35,36^ A clinical trial in Cameroon reported that VIA was inferior to cytology as a triage test among HPV-positive women,^37^ which was similar to the present study’s findings.

This trial has substantial implications for China’s national screening program. Although the national program facilitated the development of primary health care centers by improving infrastructure and personnel, the numbers of qualified cytologists and gynecologists have increased slowly.^13^ The limited health resources remain a bottleneck for the expectation to implement cervical cancer screening in 80% of the counties across China by 2022.^38^ This trial provides evidence for the advantages of hrHPV testing in detecting more precancerous lesions and cancer compared with cytology or VIA/VILI at 1 round of screening, guaranteeing a lower risk for women who test negative within 2 years. Cost-effectiveness evaluation showed that conducting low-cost hrHPV testing every 3 to 5 years in rural areas and hrHPV testing every 5 years in urban areas was cost-effective.^14,15^ Polymerase chain reaction-based hrHPV genotyping for urban areas and hybrid capture-based hrHPV testing for rural areas are effective screening modalities in primary health care centers.

The present study has several strengths. To our knowledge, it is the first randomized trial with a large population and multicenter design testing for cervical cancer screening in mainland China. The sites and clinical procedures were selected to characterize the economic and health resource status of local areas. It was embedded in the ongoing national program, and all screening was performed by local health providers, permitting an assessment of the naturalistic performance. To achieve unbiased verification of disease at the 24-month screening, co-testing or combined tests were performed, and most missed cases at baseline would have been detected. This trial was unique in that it had a large population to evaluate the performance of visual inspection as a triaging test for hrHPV-positive women.

### Limitations

First, the short time interval for the 2 screening rounds restricts the possibility of further evaluation for baseline-negative women according to the guidelines.^39,40^ Second, different HPV tests may result in within-group variation for the hrHPV arm. However, all hrHPV tests have been well verified, and the tests used in urban sites reached high agreement.^8,41,42,43,44^ Third, 9% of the women with positive baseline tests did not undergo colposcopy due to the need for extra visits to the clinic, which may result in missed cases. It is supposed that the combined screening at 24 months would have detected the majority of the missed cases. Finally, the purposive selection of the sites limits the generalizability of this study’s results to other low-income and middle-income countries with even fewer health resources.

## Conclusions

Testing for hrHPV is an effective primary screening test in primary health care centers. It is reasonable to incorporate hrHPV testing (polymerase chain reaction-based for urban areas, hybrid capture-based for rural areas) into China’s national program to meet the public health demand of its large population.

## Notes

Supplement 1. Trial Protocol

Supplement 2. eFigure 1. National Map Presenting the Locations of the Study Sites eFigure 2. Study Profile eTable 1. Baseline Characteristics and Primary Screening Results for 24-month Followed-up and Lost Follow-up Participants eTable 2. Population and Outcomes for hrHPV Testing, Cytology, or VIA/VILI Arms at 24-month Follow-up Screening and the 24-month Cumulative Results eTable 3. Estimation of Triaging HPV-positive Women with Cytology, VIA/VILI, or Direct Colposcopy eTable 4. Outcomes for hrHPV Testing Arm, Cytology Arm, and VIA/VILI Arm at the 24-month Screening

Supplement 3. Data-Sharing Statement
