# Supplementary material for: Effectiveness of High-risk Human Papillomavirus Testing for Cervical Cancer Screening in China: A Multicenter, Open-label, Randomized Clinical Trial
Source: JAMA Oncol. 2020 Dec 30;7(2):1–9. doi: 10.1001/jamaoncol.2020.6575 (PMC7774051; doi:10.1001/jamaoncol.2020.6575)
Supplement: Supplement 1. — Trial Protocol [file jamaoncol-e206575-s001.pdf]

Program number	20152014	Confidentiality	Non-confidential

Special Scientific Research Fund of Public Welfare

(Protocol)

Title: Demonstration Study of Appropriate Technique for Cervical Cancer Screening in China

Institute: Chinese Academy of Medical Science and Peking Union Medical College Hospital

Principal Investigator: Jinghe Lang

Start-End Times: January 2015 -December 2017

Protocol

1. Overview

1.1 Background

Cervical cancer ranks as the fourth most common malignant tumor among women around the world, with 85% cervical cancer occurrence in developing countries and regions.

In the year 2009, the Chinese government launched a Free Cervical Cancer Screening Program for 10 million rural women within three years. Pap smears and VIA/VILI were recommended for primary screening. Since 2012, this national screening program had expanded to cover 10 million rural women annually. However, the sensitivity of Pap smears and VIA/VILI to detect cervical intraepithelial neoplasia 2 or worse lesions (CIN2+) varies greatly due to its subjectivity. Unsatisfactory performance for Pap smear and VIA/VILI as primary methods, and lack of qualified cytologists and gynecologists are the challenges for cervical cancer screening in developing countries, especially in rural areas.

At the same time, a large number of studies have confirmed that HPV testing is effective in detection of cervical cancer and precancerous lesions. At present, commercial HPV tests are of high sensitivity and high negative predictive value, objective, reliable and repeatable, which is a revolutionary transformation for cervical cancer screening from cell morphology to molecular biology. However, most available HPV tests are expensive, require experienced personnel and high-quality lab conditions, which is difficult to be implemented in low-resource settings. HPV testing are not yet be used in screening program with large population in China.

This project is intended to explore and evaluate the clinical performance of different primary screening methods for cervical cancer in different economic level areas in China, and evaluate the cost-effective, feasibility when implement in primary care hospitals, to improve the quality of the national cervical cancer screening, and the challenges of cervical cancer screening in China. Furthermore, the study aims to provide a national guidance for cervical cancer screening.

1.2 Current Status and Future study

According to the GLOBOCAN 2012, cervical cancer ranks the third most common malignant tumor among women worldwide. Pap smears, liquid based cytology (LBC), Visual inspection with acetic acid or Lugol's iodine (VIA/VILI), colposcopy and HPV testing are the main technologies for cervical cancer screening and early diagnosis. It has been proven that well-organized cytology screening could prevent cervical invasive cancer effectively, and the morbidity and mortality of cervical cancer has been declined in developed countries. Establishing a comprehensive primary screening program with cytology in developing country is difficult due to the lack of qualified cytologists and health resource. A randomized controlled trial (RCT) in India proved that VIA can reduce the incidence and mortality of cervical cancer in areas with inadequate health resources. It was also recommended as primary screening in low-resource settings by World Health Organization (WHO).

An RCT screened 170 thousand women in European found that the incidence of the women screened by HPV based testing were 60%-70% lower than cytology-based screening, and the screening interval can be extended to five years. In some European countries, such as Netherlands, HPV testing had recommended as primary method for cervical cancer screening. In 2012, the guideline of American

Cancer Society, the American Society for Cervical Pathology and Colposcopy, and the American Society for Clinical Pathology recommends HPV testing and cytology co-testing for cervical cancer screening. The interval also can be extended to five years for women with negative results. WHO has also conducted some demonstration projects using HPV testing as a primary screening worldwide. There are many kinds of HPV testing, including HPV DNA, mRNA and early protein (E6/E7) testing. Most evidence on the clinical performance of HPV as primary screening is based on the data of HC2, which was approved by America FDA for cervical cancer screening in 2003. However, it is difficult to be implemented in low-resource settings because of the price, and requirement of experienced laboratory and high-quality lab conditions. It is imperative for Chinese scientists to explore and find an affordable, fast, simple test that could be incorporated into large population screening in rural areas of China. In 1998, scientists from the Cancer Hospital of Chinese Academy of Medical Sciences, (CHCAMS) cooperated with National Institute of Health of the U.S.(NIH), and developed an low-cost HPV test, financially supported be the Bill-Melinda Gates Foundation. This project will obtain the nationwide data of HPV testing as primary screening, and evaluate the feasibility and effectiveness of HPV testing in large-scale population-based cervical cancer screening in China.

There may be several options for primary screening of cervical cancer in future, according to the characteristics of the population, economic status, and the health resources. High-quality researches of cervical cancer screening and pilot studies have been conducted in China. However, the protocol of the national screening program needs to be updated. It is important to evaluate the performance of the current screening, optimizing the strategies, investigating the capacity of health

service system in primary care settings, conducting health economics evaluation, and developing project management standards for large population.

2. Objectives

2.1 Overall

This project aims to explore and determine the most efficient, cost-effective, and well acceptable screening program that appropriate for different economic level areas in China, optimizing the screening strategies that can be implemented nationwide.

2.2 Specific objectives

2.2.1 To explore and evaluate the short-term clinical performance of primary screening methods in real-world. Compare the accuracy, efficiency and missed diagnosis rate of the three approaches. Evaluate the performance of the different screening strategies under the basic conditions of different economic levels in China.

2.2.2 To evaluate the economic efficiency for screening arms in population-based screening, analysis the medium- and long-term effect, cost-effectiveness and cost-benefit. Explore the appropriate strategies for cervical cancer screening in China. The decision analysis model of health economics will be established to explore the feasibility of incorporating cervical cancer screening and early diagnosis into the national budget.

2.2.3 To evaluate the awareness and attitude of cervical cancer screening in different population, and investigate the potential barriers. It can provide evidence for the promotion, education and mobilization of cervical cancer screening. It can also identify potential obstacles of participation of the target women, problems

faced by health workers and police makers at all levels, and provide evidences for the government.

2.2.4 Cancer monitoring system will be established to monitor the risk factors, morbidity and mortality of cervical cancer, and observe dynamically. Training program will be provided to the local cancer registry staff, improving the quality of cancer registration.

3. Proposal

3.1 Project contents and key issues to be resolved

3.1.1 Clinical research

In the first year, enrolled women would be screened by cytology, VIA/VILI (only for rural sites) or HPV testing, respectively. In the third year, enrolled women would be screened by cytology, HPV testing or VIA/VILI (only for rural sites) simultaneously. Women with positive primary screening result would be refer to triage or colposcopy (Figure1a, 1b). The pathological results are the gold standards to determine whether treatment is needed.

3.1.2 Economic evaluation

Including establish models, field investigation and data collection. Based on the previous data from the department of Epidemiology in CHCAMS, the models would be established and adjusted. Collect the data from cancer registration, and incorporate the relevant parameters into the models to simulate the occurrence and development of cervical cancer, and to predict the medium- and long-term screening effect and costs.

3.1.3 Evaluate and compare the awareness and attitude of cervical cancer screening among different population, including the local government staff and local health workers from the counties, towns and villages.

3.1.4 Investigate the risk factors, monitor the morbidity and mortality of cervical cancer, and observe the changes. Improve the quality of cancer registration, and obtain the data of cervical cancer in local sites.

Key issues to be resolved in this study:

1. Under the conditions of primary facilities and technicians, which screening strategy is the most feasible and efficient?

2. How to get the most profit with the limited resource input? Can China's current public health system support high quality cervical cancer screening in areas with insufficient health resources and carry out it sustainably?

3. How to get more women to volunteer for screening programs? How to solve the difficulties faced by health workers and government managers? How to improve the quality of screening programs from the perspective of managers?

4. Can the existing cancer registration system effectively evaluate the medium - and long-term effects of screening programs? What are the risk factors for rural women? Has it changed and what is the trend of change?

3.2 Methods

3.2.1 Design

It is a multi-center population-based screening cohort study. Multi-stage sampling would be conducted.

3.2.2 Population

This study is embedded in the national cervical screening program of China. The inclusion criteria as follows: ①35-64 years-old; ② No history of cervical cancer or hysterectomy; ③ Non-pregnancy, without pregnant symptoms; ④ Understand the study procedures and voluntarily participated.

3.2.3 Sample size

① Primary out come

Positive rate of primary screening and CIN2+ detection rate.

② Hypothesis

The detection rates of HPV are statistically different from that of existing screening methods in real-world.

③Calculation

The detection rate of CIN2+ in the previous reported data of the national cancer screening program was 0.14%. Pilot study showed that the detection rate of CIN2+ of HPV was 0.55%. Considering that national cancer screening sites are experienced with VIA/VILI or cytology, we assume that the detection rate of CIN2+ for VIA/VILI and cytology to be 0.19%. The adjusted α level of the multiple tests was 0.025 in rural sites, the minimum sample size for each arm is 7055 providing a power of 90% to detect the difference. We assume the follow-up rate of the screened population is 80% and the sample size for each arm is 8,819. The α level was 0.05 in urban sites, the minimum sample size for each arm is 5,973 that providing a power of 90%. We assume the follow-up rate of the screened

population is 80% and the sample size for each arm is 7,467. The sample size of 63,000 in the preliminary application plan can meet the expected analysis requirements.

3.2.4 Sampling

Based on 221 national cervical cancer screening sites, multi-stage sampling would be conducted (Three-stage sampling) as follows.

① According to geographical location, the 221 national screening sites are divided into 7 regions (northeast, north, northwest, central, east, south, and southwest).
② According to the data of the national cancer registration and the local incidence of cervical cancer, 2-4 sites would be selected by purposive sampling from each geographical region, including high risk areas ($>20/10^5$), medium risk areas ($10-20/10^5$) and low risk areas ($<10/10^5$).
③ 3000 eligible women would be recruited by convenient sampling from each selected screening site.

3.2.5 Clinical research methods

The 3000 women from each site would be randomly assigned into three arms by software. The randomization program, generated by statisticians at the CHCAMS, is pre-embedded and hidden in an ACCESS registration database sent to each project site. In the first-round screening, enrolled women would be screened by HPV testing, cytology or VIA/VILI (only for rural sites). In the third year, enrolled women would be screened by HPV testing, cytology or VIA/VILI (only for rural sites) simultaneously (Figure1a,1b). All the clinical examination, sample collection and laboratory testing would be performed by local health workers.

① Cytology

It has been proven that cytology-based screening has proved to be an effective screening method for cervical cancer in developed countries, and it is also one of the optional methods in the national screening in China. All slides would be graded by a local pathologist according to Bethesda 2001 classification system, atypical squamous cells of undetermined significance (ASC-US) or worse lesion are defined as abnormal cytology.

② VIA/VILI

In developing countries, it has been proved that VIA/VILI is an effective method for primary screening, and it is also recommended as primary screening in the national program. All the clinical examination would be performed by local health workers in our program. The gynecologist will examine the cervix with naked eyes under a bright halogen focus lamp after applying 5% acetic acid for VIA/VILI, and the result would be recorded 1 minute later. Lugol`s iodine should be applied if necessary. Any abnormal of VIA or VILI would be referred to colposcopy.

③ HPV testing

The HPV test selected should have been approved by the CFDA. careHPV test has been shown in several studies to be optimal in low resource areas, and would be used in rural sites in this program. Roche cobas 4800 or Zhijiang HPV genotyping would be used as primary screening in urban sites. Women with positive results would be referred. All the clinical sample collection would be performed by local gynecologists. All laboratory work would be conducted by trained local lab technician.

④Colposcopy

Women will be recalled for colposcopy according to the primary screening or referral results. Lesion-targeted biopsies were performed. In cases of high-grade cytology abnormality but negative colposcopy findings, four-quadrant random biopsy at the squamous column junction and endocervical curettage (ECC) was performed.

⑤ Pathological diagnosis

The histopathology is the gold standard for the treatment or follow-up. Women with negative diagnosis or CIN1 would be followed-up in the second-round screening. Women with CIN2+ diagnosis will leave the program and should be treated in time. The diagnosis of the local pathologists will be reviewed be a pathologist from the supervise hospital.

3.2.6 Economic evaluation

Including establish models, field investigation and data collection. Based on the previous data from the department of epidemiology in CHCAMS, establish and adjust economic evaluation models by TreeAge Pro software. Collect the data of screening and treatment, and cost for different population by fields investigation, as well as cancer registry data. The parameters will be incorporated into the model to simulate the occurrence and development of cervical cancer of the three arms, and predicting the medium and long-term screening effect and the costs.

By estimating the incidence, mortality, and the cumulative effects such as lifetime risk of cervical cancer, and lifesaving costs and incremental cost, under the guidance of the current national screening program, including the age range and interval, evaluating the best strategy, thus providing evidence for nationwide cervical cancer.

3.2.7 Cognitive survey

To investigate the cognition of the screening population on cervical cancer screening, and to compare the attitude on different screening methods of the screened population. Besides, we will investigate the cognition and attitude of the local government of county, township and villages, and the local medical workers. To investigate the reproductive status of women China, to evaluated the cognition and attitude of screened population and the organizer, to recognize the potential obstacles of large-scale population screening, and to find the solutions, so as to provide evidences for police makers.

3.2.8 Risk factors monitoring of, morbidity and mortality of cervical cancer registry

The population risk factors in the selected screening sites will be monitored by investigator. A questionnaire is designed for investigating the risk factors of cervical cancer. Trained doctors will followed-up the screened population to investigate the exposure to environmental risk factors. The project plans to further improve the cancer registration system at the study site, so as to obtain a complete and accurate information of the incidence and mortality of cervical cancer.

3.2.9 Data management

Visual FoxPro will be used for data management. All data will be double-input in the database by separated trained investigators and double-checked. SPSS, SAS or other statistical software were used to analyze qualitative and quantitative indicators. The Markov model was established by using TreeAge software to simulate the natural history and history of cervical cancer and calculate the

economic evaluation indexes such as QALYs and DALYs. All statistical tests were double-sided at the 0.05 level.

3.2.10 Evaluation indicators and methods

The clinical data and survey data obtained from the study sites will be used to evaluate the efficiency of the screening, the health economics indicators, and the cognition and attitude status. Detailed calculation indicators and analysis methods are as follows:

① Analysis of the clinical screening data

Indicators: positive rate of the three primary screening methods, CIN2+ detection rate, positive predictive value and early diagnosis rate; false negative rate and negative predictive value. The incidence and mortality of cervical cancer in the areas during the program will be tracked using the average of cancer registration data in the 2 years prior to the program as the baseline.

Positive rate: the proportion of women, who need to be referred for colposcopy according to the definition of the study.

CIN2+ detection rate: the proportion of women with pathologically confirmed CIN2+ in the primary screening arms out of the screened women in each arm.

Positive predictive value: according to the definition, the proportion of women with pathologically confirmed CIN2+ lesions out of the number of positive primary screening.

Early diagnosis rate: the proportion of CIN2, CIN3/ in-situ cancer and early cancer among all CIN2+ lesions in the three groups of screened women.

False negative rate: percentage of women who were screened negative in the first-round screening and are detected with CIN2+ in the second-round screening.

Negative predictive value: the proportion of women who are negative in the second-round screening.

Statistical analysis methods: chi-square test was used for comparison of categorical data, and LSD, Bonferroni and other methods were used for multiple comparisons. Quantitative data will be analyzed by t test, variance analysis and so on.

② Health economics evaluation

The data source including a 16-year cohort study conducted by the Department of Epidemiology, CHCAMS, data from the "The Eleventh Five-year Plan" of the national science and technology, and other government supported health economics evaluation studies, on site investigation and literature review.

A. Cost-effectiveness analysis: the reduced cases (CIN2, CIN3, and cervical cancer) in the three screening arms, the number of reduced cervical cancer deaths, and the reduced cost of avoiding one death for each of the screening arms. The HPV testing arm is the evaluation group, and the VIA/VILI or pap smear are the control.

B. Cost-utility analysis: increased quality adjusted life years (QALYs), saved disability-adjusted life years (DALYs), and cost-utility ratio.

C. Cost-benefit analysis: the indicators of cost-benefit analysis are cost (C) benefit (B) ratio.

③ Analysis of cognition and attitude survey data

The knowledge, feelings and attitude towards cervical cancer screening (stratified by age, education level, occupation, etc.) will be compared. To investigate the awareness of cervical cancer screening among health workers (maternal and child care system and other primary health care system) and government policymakers (department of science, education, and health, or other government departments.

Statistical analysis methods: chi-square test was used for comparison of categorical data, and LSD, Bonferroni and other methods were used for multiple comparisons. Quantitative data will be analyzed by t test, variance analysis and so on.

3.2.11 Informed consent and ethical issues

Approval from the ethics committee of Peking union medical college hospital, Chinese academy of medical sciences will be obtained before the enrolment, and all participating hospitals should approve the study as well. All enrolled women will be screened with one of the primary screening approaches free of charge. Women will be adequately informed of the benefits and harms of participating in the study, completely voluntary, and they could drop out of the study at any time. Women could only participate in the study after signing the informed consent.

3.2.12 Quality control

The current established site from the national screening program will be selected as the study sites. With experiences of field work, primary screening sites carry out screening work under the guidance of superior hospital. The followings are details of quality control:

① Before launch of the study, the research team shall formulate and finalize working plan, operation manual, questionnaire and other management documents.

Health providers at local should be trained by experts from PUMCH on gynecological examination, cytopathology, histopathology, laboratory testing, and data management, to ensure the local investigators have adequate training that they could totally understand the requirements of the study.

② The results of cytology at local primary sites will be reviewed by an expert from the supervising hospital at provincial level and all ASC-US+ and 10% of NILM will be selected randomly and reviewed at the baseline and the 24-month screening.

③ Histopathological diagnosis is the gold standard for the case confirmation. The pathological slides shall be read by the local doctor, and all positive slides and 10% of the slides diagnosed as negative shall be reviewed by an expert from the supervising hospital at provincial level.

⑤ A team for data management will be responsibility for quality control of the data. The questionnaires and the clinical data of the study should be stored separately in assigned room in the study site. Only the data manager, entry clerk, investigator, and laboratory staff may have access to the original data. The data entry should be finished within one week. Entry clerk has the right to read and input data, only the data manager has the right to modify the entered data. The administrator, entry clerk, principal investigator will have the password of the database. In order to ensure the accuracy of the input data, all data will be double-entry and logically-checked. Any inconformity will be check and revise according to the paper record.

4. Innovation

This study has the following innovations:

4.1 It is the first time for introducing HPV testing into the national screening program in China on a large-scale population-based, multi-center screening study, and compared with the current primary screening methods;

4.2 All screening procedures of this study will be performed by the health providers from primary care hospitals, which makes it able to evaluate the performance of the different screening approaches in real-world.

4.3 Establishing monitoring system for cervical cancer risk factors and improving the cancer registration system to obtain the incidence and mortality of cervical cancer in rural areas at the study sites.

4.4 This program will lay a foundation for the establishment of a long-term and systematic evaluation system by improving the cancer registration system.

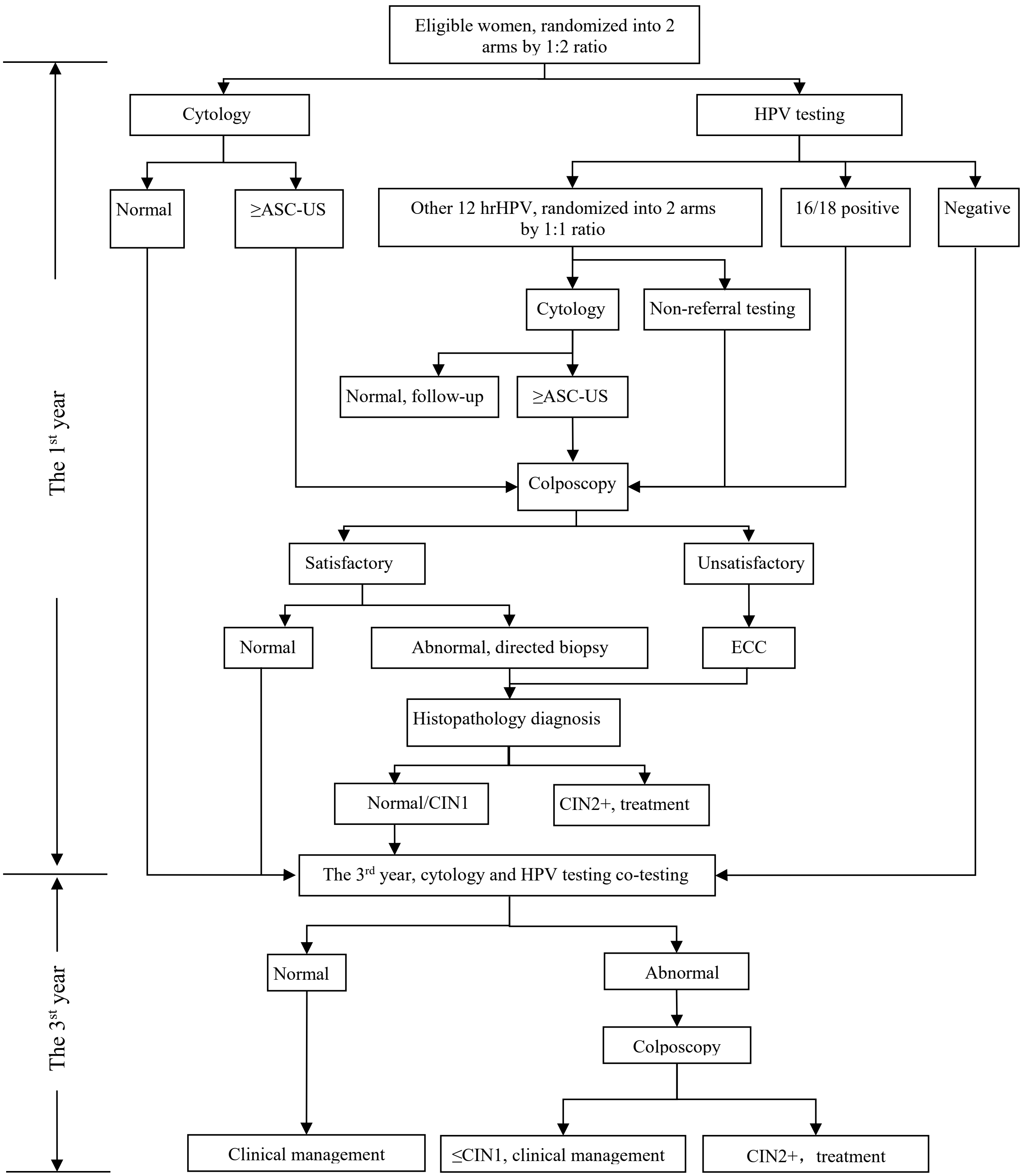

Figure 1a. Flowchart of Clinical Study in Urban Sites

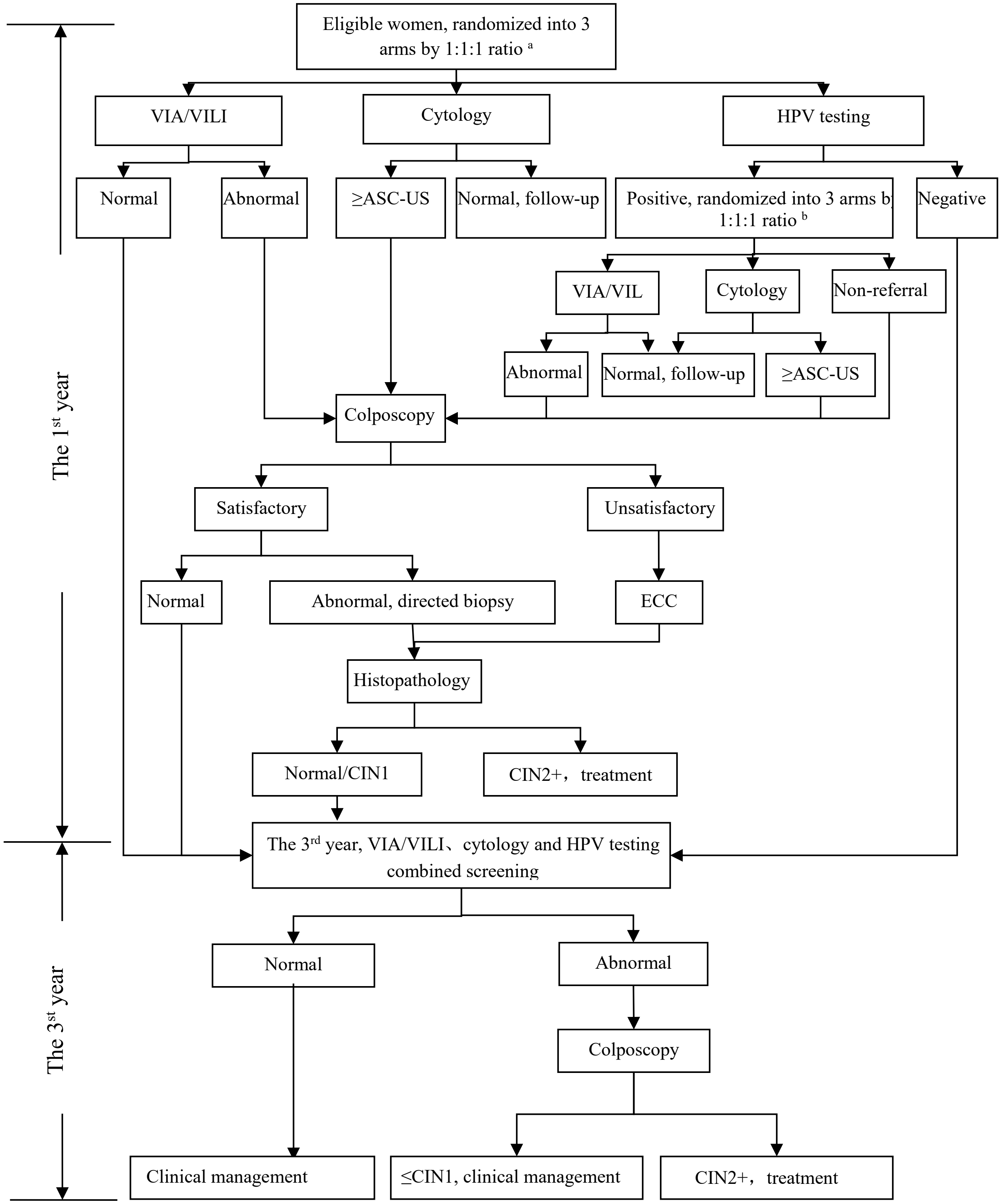

Figure 1b. Flowchart of Clinical Study in Rural Sites

a. Areas without cytological ability are randomly divided into HPV testing or VIA/VILI arm by 2:1 ratio; b. Areas without cytological capacity are randomly divided into VIA/VILI-triage or direct colposcopy by 1:1 ratio.

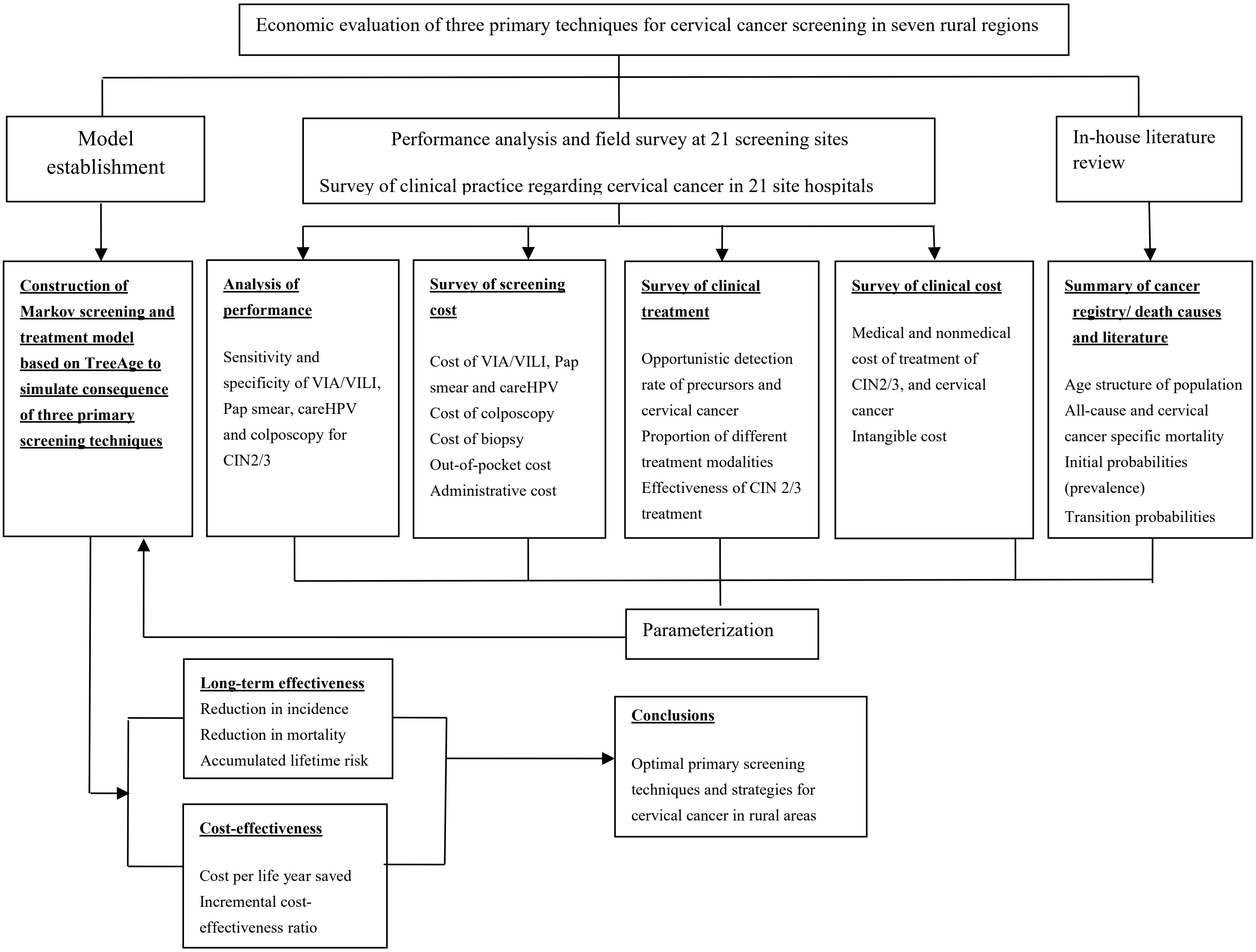

Figure 2 Diagram of Economic Evaluation of Cervical Cancer Screening Techniques and Algorithms

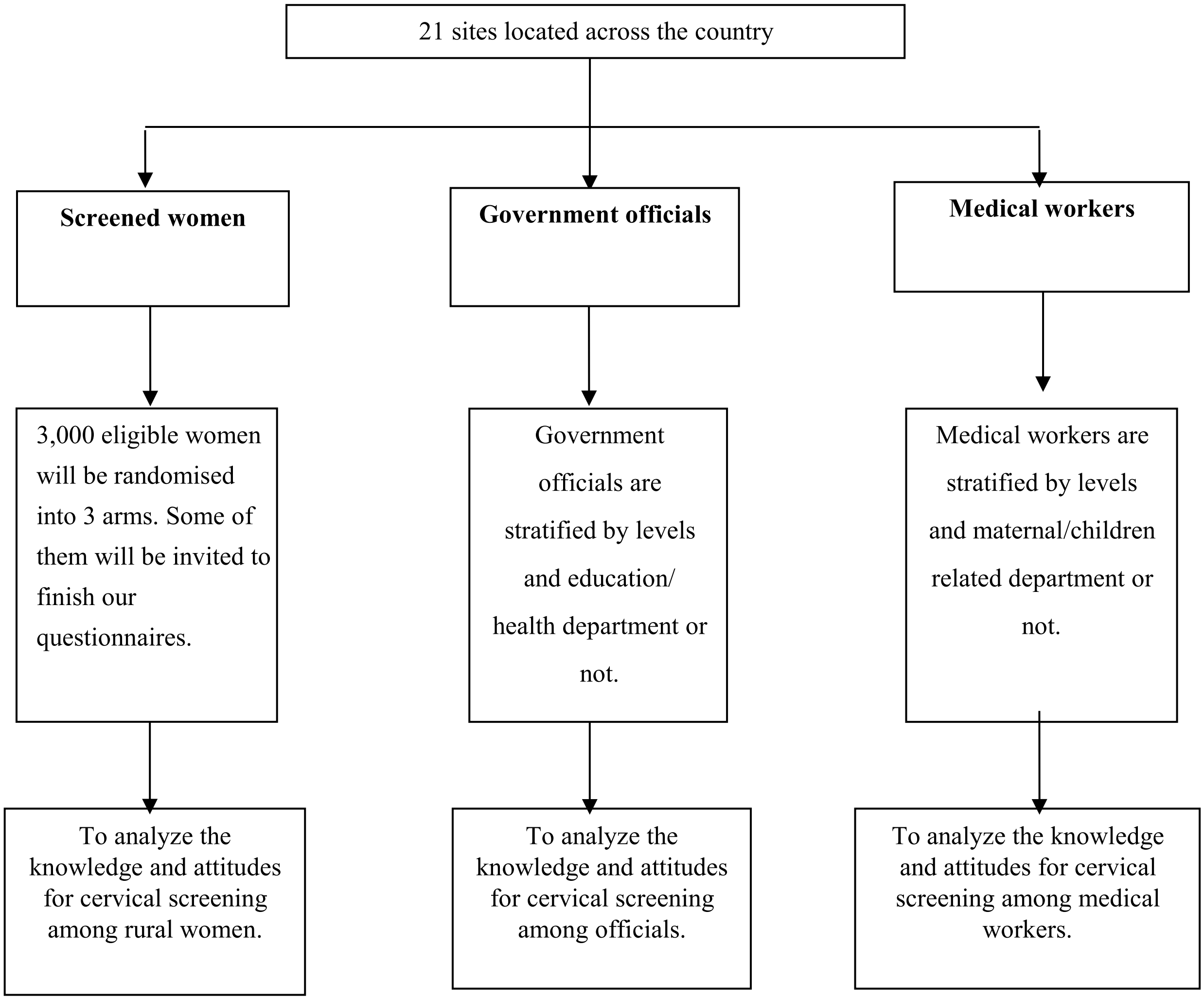

Figure 3 Knowledge and Attitude Investigation

项目编号	20152004	密级	公开

公益性行业科研专项项目
（技术方案）

项目名称：适合中国农村地区的宫颈癌筛查技术与示范研究

项目承担单位：中国医学科学院北京协和医院

项目负责人：郎景和

起止年限：2015 年 1 月至 2017 年 12 月

一、项目概述

（一）立项依据

2009 年，我国政府启动在农村实施“两癌”检查项目，在 3 年内为全国 1000 万女性筛查宫颈癌。2012 年筛查的范围扩大到了每年 1000 万妇女。所采用的方法为巴氏涂片（Pap smear）或醋酸/碘染色肉眼观察（VIA/VILI）巴氏涂片漏诊率高，而 VIA/VILI 的假阳性和漏诊率都较高，这两种技术在实际筛查应用中结果尚不能令人满意，是目前我国宫颈癌筛查扩大工作遇到的亟待解决的瓶颈。如此大规模的人群筛查，需要科学的适宜筛查技术和完善的质量管理体系。

国内外大量有关宫颈癌筛查方法效果的评价研究已经证实，HPV 检测已是国内外公认发现宫颈癌和癌前病变的有效手段。目前成熟的 HPV 检测方法具有灵敏度高和阴性预测值高的技术特点，并且具有客观、短时间内可获得结果、可靠、易于重复等优势，可以大幅度减少细胞学阅片工作量，延长筛查间隔周期，提高筛查的效率，大大优于细胞学和 VIA/VILI，是宫颈癌筛查从细胞形态学向分子生物学的革命性转变。此类技术已经在部分发达国家和包括我国在内的许多发展中国家的城市地区陆续采用，但在我国大规模人群筛查项目中尚未推广。

本项目旨在探索和评价适合我国不同经济水平农村地区宫颈癌筛查和早诊技术，符合成本效益的筛查方案，更加可行、高效、易被接受的筛查机制，为提高宫颈癌检查的质量和效果，解决目前筛查扩大工作遇到的巨大挑战，提升大规模人群筛查中的筛查水平和质量，建立科学评价筛查方案的体系，指导全国性农村地区宫颈癌的筛查，保障广大农村妇女的健康，具有极强的现实意义。

（二）国内外研究现状与发展趋势

据世界卫生组织 GLOBOCAN 2012 数据显示，宫颈癌为全球女性肿瘤中第三大常见恶性肿瘤。国内外现有的宫颈癌筛查与早诊技术主要包括传统巴氏涂片、液基细胞学、VIA/VILI、阴道镜检查及 HPV 检测。发达国家的经验表明，通过大规模、有组织的细胞学筛查可以大大降低宫颈癌的发病率和死亡率。由于卫生资源的缺乏，发展中国家难以建立有效、成熟的细胞学筛查系统。印度的一项随机对照研究发 现，通过 VIA 筛查可以降低卫生资源不足地区的宫颈癌发病率和死亡率。世界卫生组织（WHO）也在其筛查指南中推荐在欠发达地区采用 VIA 筛查宫颈癌。

欧洲一项对 17 万妇女的随机对照研究结果显示，以 HPV 检测为基础的筛查比以细胞学为基础的筛查妇女宫颈癌的发病率低 60%-70%，并可将筛查间隔延长到 5 年。在部分欧洲国家，如荷兰，已将 HPV 检测作为官方推荐的初筛方法用于宫颈癌的筛查。2012 年，由美国癌症协会、美国宫颈病理及阴道镜协会、美国临床病理协会联合推出的宫颈癌预防及早诊的筛查指南中，明确推荐采用 HPV 检测与细胞学联合筛查（Co-testing），对联合筛查阴性的妇女，筛查间隔同样可延长至 5 年。WHO 也在世界范围内开展了以 HPV 检测技术作为宫颈癌初筛方法的示范性项目。HPV 检测技术种类较多且比较成熟，主要包括对 HPVDNA、mRNA、早期蛋白（E6/E7）等生物标志物的检测。目前，使用较为广泛的 HPVDNA 检测技术为 HC2（Hybrid Capture II），该技术早在 2003 年就获得美国 FDA 批准可用于宫颈癌的人群筛查。然而，HC2 检测价格昂贵、对实验条件要求较高，并不适用于低资源地区的人群筛查。因此，找到一种适宜在中国农村地区大规模人群宫颈癌筛查中应用的，快速、准确、简便的初筛技术是我国宫颈癌防治领域专家共同的努力方向。早在 1998 年，中国医学科学院肿瘤医院的团队

就与美国合作开展研究，探索适合我国国情的宫颈癌筛查技术。由美国 NIH、比尔-梅琳达盖茨基金会支持的多个国际合作项目，成功研发并证实了在中国农村地区采用 HPV 检测技术筛查宫颈癌的有效性。通过本项目的开展，可获得全国性的数据，对采用 HPV 检测进行大规模人群宫颈癌筛查的可行性及有效性等指标进行综合评价。

宫颈癌筛查策略的发展趋势是倡导在一个国家或地区内多种筛查技术并存，根据人群或地区的特点，开展多样的筛查项目，将筛查技术推广到适合地区。我国有优秀的宫颈癌筛查技术研究及其相应试点的工作，但尚无成熟的全国性宫颈癌筛查项目管理规范。因此，对不同筛查方法、全国多个卫生资源不足地区的筛查进行绩效评价、开展 卫生经济学研究以及制订大规模人群项目管理规范至关重要。

二、项目目标

本研究总体目标：确定适合我国国情，适宜在不同经济水平地区使用的高效、符合成本效益、妇女可接受的最优筛查方案，制订可全国推广的筛查方案并为项目质量管理提供范本。

分目标：

1.探索在基层设施、技术人员等条件下，采用不同初筛技术的近期筛查效果。比较不同技术的初筛效率和漏诊率，评价在我国不同经济水平地区的基层条件下，符合实际情况的高效筛查方案。

2.评价不同初筛技术应用于大规模筛查项目的卫生经济学效果，分析不同筛查方案的中远期效果和成本效果、成本效益，筛选出适合我国基本国情的筛查方案；建立卫生经济学决策分析模型，从供、需双方探讨将宫颈癌筛查及早诊方案纳入国家医疗保障体系筹资方案的可行性。

3. 评价不同人群对宫颈癌筛查的认知及态度，了解开展人群筛查及推行不同筛查方案的潜在障碍。认知的调查研究可以为宫颈癌筛查的宣传教育及动员提供有力依据，同时能发现妇女参与筛查的潜在障碍、卫生工作者及各级管理者工作中面临的问题，为政府决策者制订全国性筛查项目管理标准提供理论依据。

4. 在本研究项目点开展农村地区肿瘤监测工作，对宫颈癌危险因素及肿瘤发病及死亡进行监测，动态观察其变化。培训专职工作人员，完善肿瘤登记，为后期建立长效的评价体系奠定基础。

三、研究方案

（一）主要研究内容与拟解决的关键问题

1. 临床研究：分别（第一年）或同时（第三年）采用细胞学、VIA/VILI、HPV 检测三种技术对随机分组的妇女进行初筛，筛查结果阳性的妇女如流程图 1a、1b 所示进行进一步检查或直接转诊阴道镜。最终以阴道镜下病理活检结果判断妇女是否需要治疗或随访观察。

2. 卫生经济学评价：包括模型构建、现场调查和数据收集工作。基于中国医学科学院肿瘤医院流行病室前期工作基础，构建和调整模型。收集肿瘤登记和死因回顾调查数据，将相关参数纳入模型，模拟宫颈癌的发生发展，预测中远期筛查效果和相应成本费用。

3. 对筛查人群对宫颈癌筛查的认知及态度进行问卷调查，比较调查对象对不同筛查手段的认知和态度。同时，调查县、乡、村三级政府部门工作人员和医务工作者对筛查项目的认知和态度。

4. 在当地开展有效的肿瘤危险因素监测，对危险因素进行调查，掌握人群宫颈癌危险因素的动态变化情况。通过进一步完善肿瘤登记获得该地区妇女宫颈癌的发病率及死亡率。

本研究拟解决的问题：

1. 在我国基层设施、技术人员等条件下，采用何种筛查方案最可行、最高效？

2. 如何用有限的资源投入获得最大的收益？我国现行的公共卫生保障体系能否支撑卫生资源不足的地区进行较高质量的宫颈癌人群筛查，并可持续进行？

3. 如何让更多的妇女自愿参与到筛查项目中？如何解决卫生工作者及政府管理人员面临的困难？如何从管理者角度提高筛查项目的质量？

4. 现有的肿瘤登记系统是否能够有效地评价筛查项目的中远期效果？农村妇女的危险因素分别情况？是否发生变化以及具有怎样的变化趋势？

（二）研究方法

1.研究设计：

本研究为多中心人群筛查队列研究。为保证研究对象的代表性，采用多阶段抽样方法。

2.研究对象：来源于我国“两癌”检查项目地区，入组条件如下：①年龄为 35-64 岁妇女；②无宫颈癌疾病史，宫颈完整；③无临床怀孕可疑症状；④理解研究程序，自愿参与。

3.样本含量估计：

① 主要评价指标：初筛阳性率和 CIN2+检出率。

② 研究假设：在基层实际筛查中，HPV 检测法的检出率分别与现有筛查方法的检出率之间存在统计学差异。

③ 计算方法：前期国家“两癌”筛查项目的 CIN2+检出率为

0.14%。前期预试验结果显示HPV的CIN2+检出率为0.55%。考虑到“两癌”筛查项目点对VIA/VILI和细胞学筛查有一定经验，假设本研究中VIA/VILI和细胞学的CIN2+检出率为0.19%。农村地区考虑多重检验调整检验水准为0.025,在检验效能为90%的条件下，HPV与VIA/VILI和细胞学比较所需样本量为各组7055人。假设研究的召回访视率为80%,各组计划入组8819人。城市地区检验水准为0.05,在检验效能为90%的条件下，HPV与细胞学比较所需最低样本量为各组5973人。假设研究的召回访视率为80%,各组计划入组7467人。因此前期申请计划中6.3万的样本量能改满足预期分析要求。

4.抽样方法：

本研究的抽样框架是全国221个“两癌”筛查项目点，采用多阶段抽样方法（三阶段抽样），具体如下：

第一阶段：按地理位置将221个国家两癌筛查项目点所在地区分为7大区（东北、华北、西北、华中、华东、华南、西南）；

第二阶段：结合全国肿瘤登记数据，充分考虑该地的宫颈癌发病水平，每个大区采取方便抽样的方法抽取2-4个项目点，共21个项目点，包括宫颈癌的高发区（＞20/10万）、中发区（10〜20/10万）和低发区（＜10/10 万）。

第三阶段：在选定的“两癌”检查项目点，采用方便抽样的方法选择3000名合格妇女。

5. 临床研究方法：

对各项目点3000名参与筛查的合格女性在基线筛查时进行随机分组。随机分组程序由中国医学科学院肿瘤医院的统计人员生成，预先嵌入并隐藏在发送到每个项目点的ACCESS登记数据库中。不同经济发展地区根据不

同分组在第一年采用不同初筛方法进行宫颈病变初筛；第三年多种方法联合使用，对基线入组妇女再次筛查（详见图1a,1b）。所有的筛查工作由基层单位负责执行。

（1）细胞学检查（Pap Smear）：发达国家的筛查实践证实为有效的宫颈癌初筛方法，也是我国“两癌”检查中可选方法之一。本研究中，所有细胞学检查均由当地医生操作，包括取材、涂片、固定、染色。采用2001 Bethesda 分类系统报告，以未明确意义的非典型鳞状细胞（ASC-US）为界值。诊断为≥ASC-US 的妇女进行阴道镜检查。

（2）醋酸/碘染色肉眼观察（VIA/VILI）：大量发展中国家的实践证实为有效的宫颈癌初筛方法，也是我国“两癌”检查初筛方法之一。本研究中，VIA/VILI 检查均由当地医生操作，涂抹 5%的醋酸溶液于宫颈表面，1 分钟后在 100 瓦的白炽灯光下观察。VIA 检查正常者做 VILI 检查。VIA 或 VILI 检查异常者进行阴道镜检查。

（3）HPV 检测法：拟选用 CFDA 批准的 HPV 检测技术。在经济水平较差的地区采用careHPV检测技术，该技术经多项研究证实适用于资源匮乏地区，目前WHO在全球发展中国家进行该技术的推广。本研究中，所有HPV检测均由当地医生采样，经培训的当地技术人员进行实验室检测。在经济水平较好的地区采用罗氏 cobas 4800 或之江 HPV 检测技术用于初筛。初筛结果阳性的妇女进行进一步分流或转诊阴道镜。

（4）阴道镜检查：上述筛查阳性妇女转诊阴道镜，由当地医生完成。阴道镜检查不满意时，行宫颈管搔刮术（ECC）。阴道镜检查满意时，如阴道镜下可疑低度或高度病变，进行阴道镜指示下活检；细胞学结果为AGC,ASC-H或≥HSIL时，同时进行ECC；如阴道镜检查为正常，根据相应临床规范处理。

（5）病理阅片：金标准，作为妇女是否需要治疗或随访观察的判断

标准。病理组织学诊断为阴性或 CIN1 的妇女进行随访观察；对组织学诊断结果≥CIN2 的妇女，应及时治疗并随访。为保证临床研究诊断的准确性，由项目点医院制片，当地病理医生及上级医院病理专家共同诊断。当诊断不一致时，以病理专家诊断结果为准。

6. 卫生经济学评价研究方法

卫生经济学评价包括模型构建、现场调查和数据收集工作。基于中国医学科学院肿瘤医院流行病室前期工作基础，利用 TreeAge Pro 软件构建和调整宫颈 HPV 动态传播模型、宫颈癌自然史模型、宫颈癌筛查、诊断和治疗干预模型。通过现场调查获得不同组人群筛查/治疗相关临床资料和成本数据，同时收集肿瘤登记数据和死因回顾调查数据。将相关参数纳入模型，模拟三组人群在不同初筛干预下宫颈癌的发生发展，预测宫颈癌中远期筛查效果和相应成本费用。

研究通过模型确定不同筛查方案下子宫颈癌发病率、死亡率和累计终生发病风险等效果指标，以及单位挽救生命年费用、增量成本效果比等卫生经济学指标，在国家推荐筛查起始年龄、筛查间隔和终止年龄下推算最佳筛查方案，从而为农村地区大范围推广子宫颈癌筛查提供科学的决策依据。

7. 认知情况调查研究方法

调查筛查人群对宫颈癌筛查的认知情况，比较调查对象对不同筛查手段的认知和态度。同时，从决策分析角度出发，搜集筛查人群、政府工作人员及医务人员对宫颈癌筛查的认知和态度数据。获得中国农村地区女性人口学及生殖健康相关信息，了解项目开展单位的基本情况，访谈卫生主管部门行政管理人员、医疗机构负责人等，了解目前筛查工作面临的问题与难点，以及推行各种筛查方案的潜在障碍，为国家宫颈癌筛查项目的推广提供科学依据；真实反映筛查对象及筛查实施者的认知态度，了解在中

国农村地区开展大规模人群筛查工作的潜在障碍，并研究其解决办法，为政府决策者提供理论依据。

8. 宫颈癌危险因素监测，发病率及死亡率监测

采用定群研究的方法，对21个筛查点的人群危险因素进行动态监测。针对宫颈癌危险因素设计调查表。经统一培训的调查员对研究队列人群随访，调查该人群的环境危险因素的暴露情况，掌握该人群宫颈癌危险因素的动态变化。

肿瘤登记可以为肿瘤相关研究提供基础资料，促进筛查工作的顺利开展。因此， 本项目计划在研究项目点当地进一步完善肿瘤登记系统，从而完整、准确地获得地区妇女宫颈癌的发病率及死亡率。

9. 数据管理

采用 Visual FoxPro 数据库软件编制的数据库进行录入和管理。所有资料由已培训的技术员完成两遍录入，由数据管理员进行核查，整理形成最终数据库。使用 SPSS、SAS 等统计软件分析定性、定量指标。应用 TreeAge 软件建立 Markov 模型，模拟宫颈癌的自然史及发病史，计算质量调整生命年（QALYs），失能调整生命年（DALYs）等经济学评价指标。所有统计检验都采用 0.05 水平的双侧检验。

10. 评价指标及方法

现场筛查获得的临床数据及调查资料将用于不同方法筛查效率的评价、卫生经济学指标的计算、认知及态度的评价。详细计算指标及分析方法如下：

（1）临床筛查数据分析：

计算指标：三种初筛方法的阳性率、对 CIN2 及以上病变 (CIN2+) 的检出率、阳性预测值以及早诊率；评价三种方法用于初筛的漏诊率以及阴性预测值；以项目启动前 2 年肿瘤登记数据的平均值为基线，

追踪项目实施期间该地区宫颈癌的发病率及死亡率。

阳性率：根据本项目方案定义初筛为阳性，需要转诊阴道镜的妇女占各组妇女人数的比例。

CIN2+病变检出率：初筛妇女中病理确诊为 CIN2+的例数占各组妇女人数的比例。

阳性预测值：根据本项目方案定义初筛为阳性的妇女中，病理诊断为 CIN2+病变的妇女的比例。

早诊率：筛查妇女中发现的 CIN2、CIN3/原位癌、早期癌占 CIN2+病变的比例。

漏诊率：第一轮筛查阴性的妇女中，第二轮筛查发现宫颈病变妇女所占的比例。

阴性预测值：初筛为阴性的妇女中，第二轮筛查仍为阴性妇女所占的比例。

统计分析方法：率的比较采用卡方检验，多重比较采用 LSD，Bonferroni 等方法进行调整。定量资料分析采用 t 检验，方差分析等检验方法。

（2）卫生经济学分析：

数据来源包括中国医学科学院肿瘤医院流行病室随访 16 年队列研究数据，国家科技支撑计划“十一五”课题和卫生部“行业专项课题”的卫生经济学评价数据资料，本项目现场调查及文献查阅等。

1）成本效果分析：比较不同筛查组减少的疾病（CIN2，CIN3，宫颈癌）发病例数，减少的宫颈癌死亡例数，不同筛查方案每避免一例死亡的成本。以 HPV 检测组为评价组，VIA/VILI 或细胞组为对照，比较新型初筛技术与传统初筛技术的增量成本效果比（ICER）。

2）成本效用分析：不同筛查方案增加的质量调整生命年(QALYs)，

挽救的失能调整生命年(DALYs),成本效用比(CUR:△成本/△QALY)。

3）成本效益分析：成本效益分析指标即成本（C）效益（B）比。

（3）认知及态度调查数据分析：

不同水平（年龄、受教育程度、职业等）妇女的认知，对不同初筛方法的主观感受以及影响因素等指标进行分组比较。计算卫生工作者（妇幼系统、非妇幼系统）及政府管理人员（科教卫生部门、非科教卫生部门）对宫颈癌筛查的认知率，分类分析态度等问题。

统计分析方法：率的比较采用卡方检验，多重比较采用 LSD，Bonferroni 等方法进行调整。定量资料分析采用 t 检验，方差分析等检验方法。

11. 知情同意及伦理问题

在项目实施前，需获得中国医学科学院北京协和医院和合作单位伦理委员会的批准。本项目中所有研究对象都会进行筛查，不设空白对照组。妇女参与研究前必须明确告知其参加本研究的利益和风险，严格遵循自愿原则，且可随时选择退出研究，妇女在签署知情同意书后方可参与研究。

12. 项目的质量控制

本研究选择国家“两癌”检查项目点开展工作，具备一定的现场工作基础，基层筛查单位均有负责单位进行指导。为进一步确保本项目的质量，我们将针对各个环节采取相应质控措施：

（1）项目启动前，项目组制定统一的执行方案、操作手册、调查问卷等管理文件，便于统一标准。项目组成立妇科检查、细胞病理学、组织病理学、实验室检测及数据管理等专家小组，对现场工作人员进行充分培训，使其充分掌握本项目的要求，考核合格后方可开展相应的工作。

（2）由负责单位对基层单位的细胞学诊断进行质控。第一年和第三年的初筛中，基层单位诊断为阳性的细胞学片以及随机抽取 10%的阴性片由上级负责单位进行复核。

（3）组织病理学诊断为本项目最终病例确认的金标准。按项目要求，病理诊断由当地医生首次诊断，所有阳性诊断的病例及随机抽取 10%诊断为阴性的玻片由负责单位进行复核，完成双人诊断病理记录。

（4）为保证研究数据的准确性，将成立数据管理组。筛查现场的研究数据表与临床处理数据表独立存放于指定房间。仅数据管理员、录入员、调查员和实验员可使用原始资料。对数据库加密，每周完成前一阶段的数据录入。录入员有权读取和录入数据，仅数据管理员有权修改已录入的数据。数据管理员、录入员、主要研究者持有数据库密码。为保证数据真实及准确性，所有资料实行双人双录及逻辑核查，出现差异的地方参照原始资料进行修改。

（三）研究创新性

本研究项目具有以下创新性：

1. 国内首次采用 HPV 检测技术在全国多中心开展大规模人群筛查研究，并与两种现有检测技术的筛查效果进行同期比较；

2. 本研究的具体实施依靠“两癌”筛查项目点的基层技术力量、基础设施等资源，可评价在现实条件下不同方法筛查宫颈癌前病变及宫颈癌的效率，具有极强的现实意义；

3. 在筛查点建立农村地区肿瘤危险因素的监测系统，监测人群中宫颈癌危险因素的变迁；进一步完善肿瘤登记系统，获得地区宫颈癌的发病率及死亡率。

4.本项目通过完善肿瘤登记系统,为建立长效、远期、系统的评价体系奠定基础。

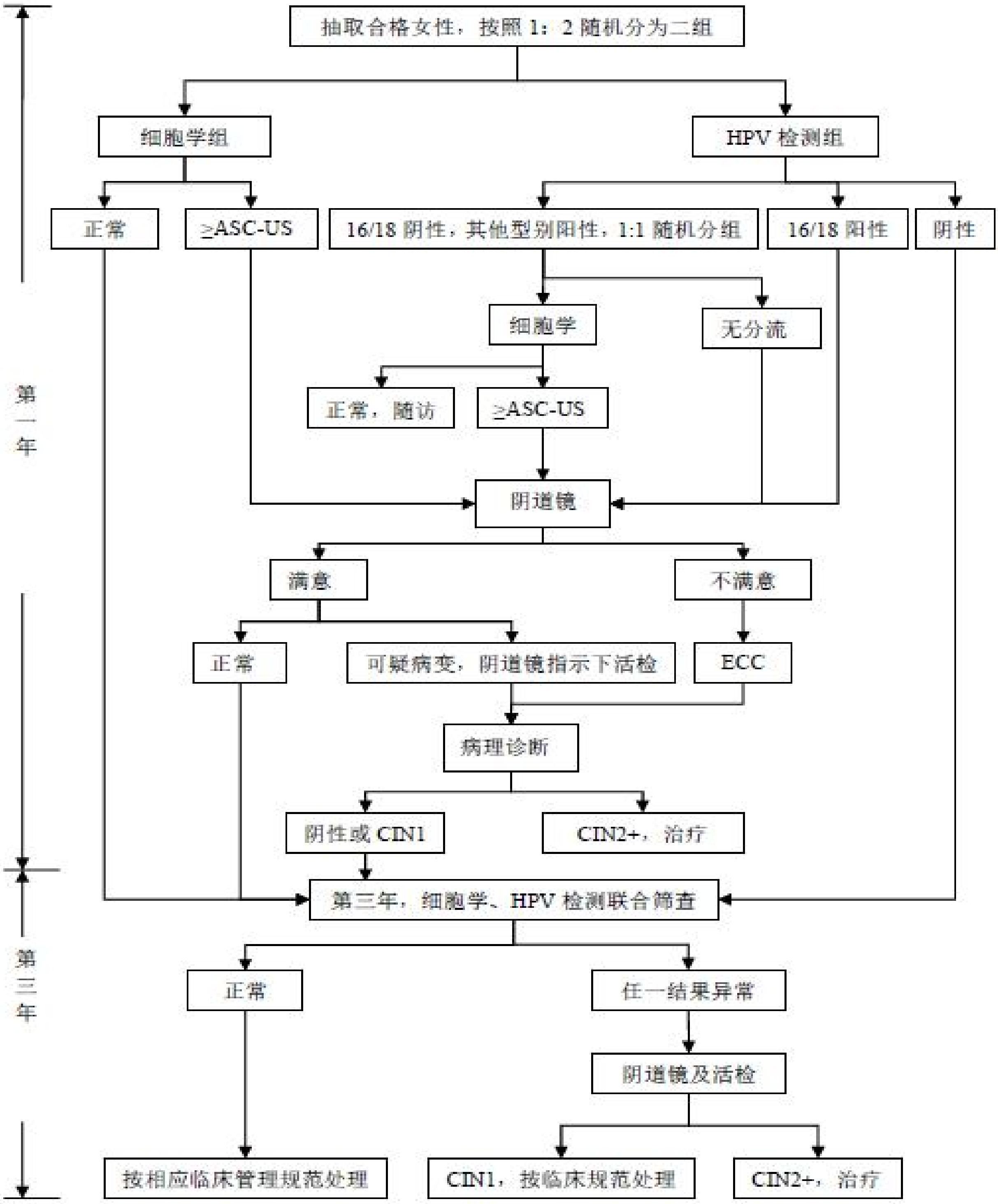

图 1a 城市地区临床技术路线

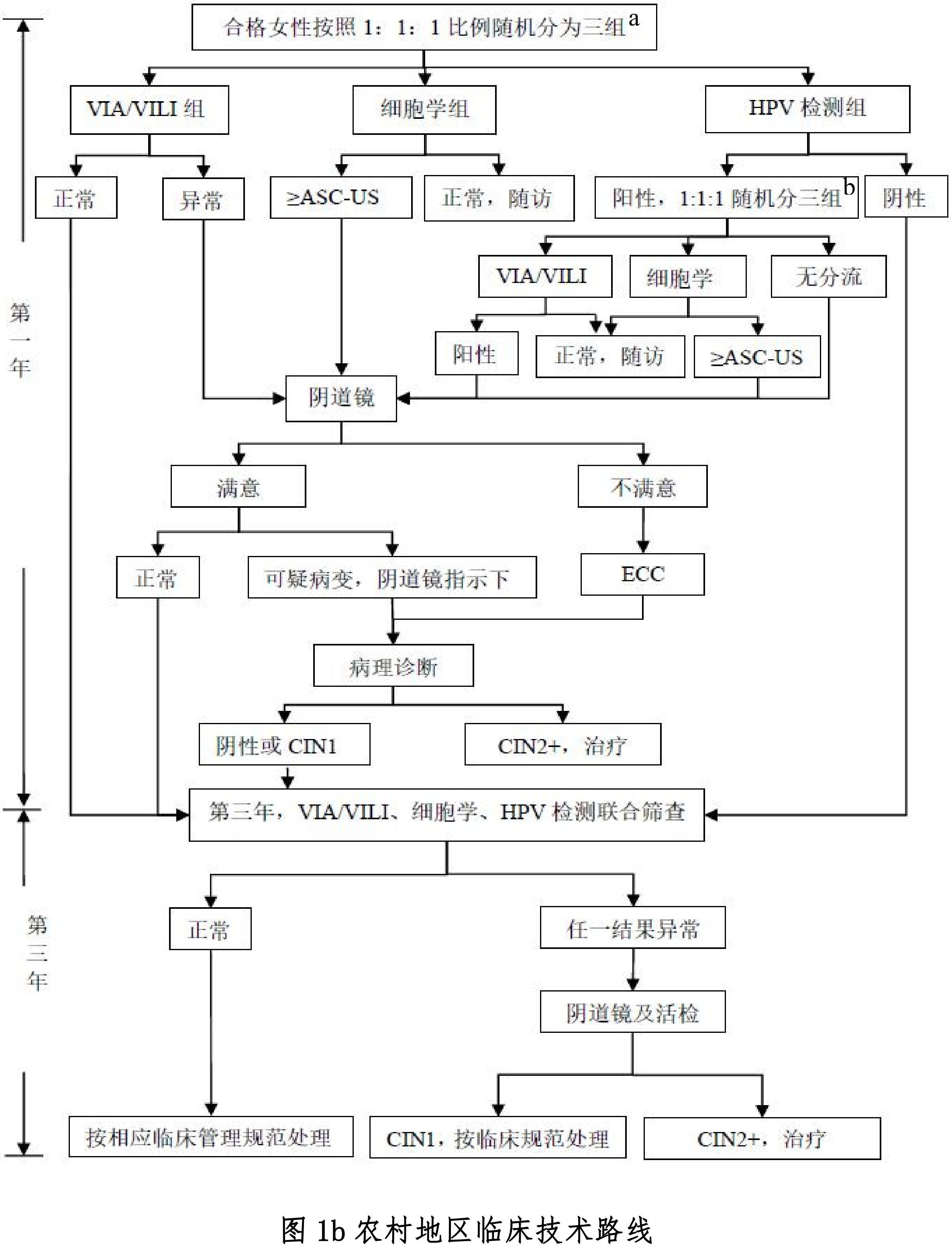

图 1b 农村地区临床技术路线

a:无细胞学检查能力地区按照 2:1 的比例随机分为 HPV 检测组或 VIA/VILI 组;
b:无细胞学检查能力地区按照 1:1 的比例随机分为 VIA/VILI 分流或不分流直接转诊阴道镜检查。

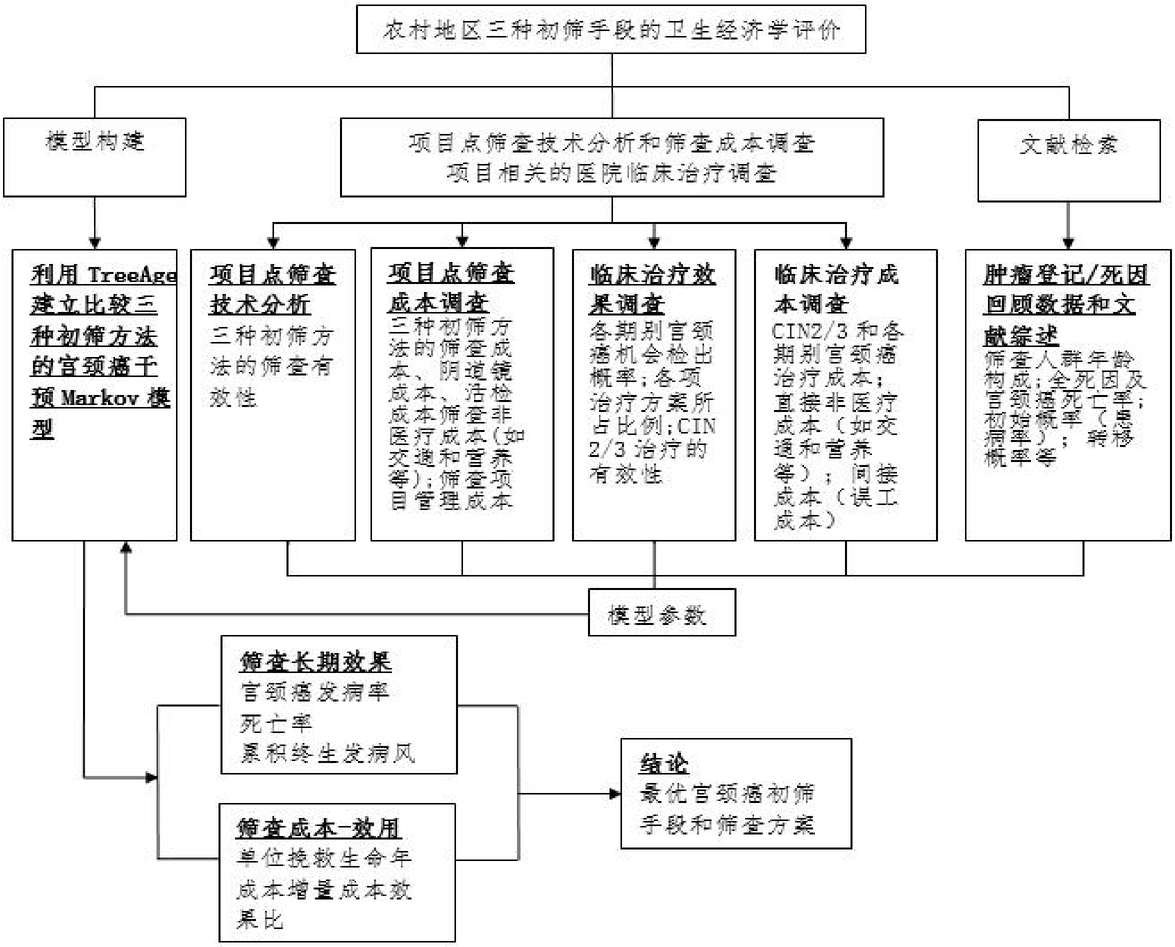

图 2 宫颈癌筛查卫生经济学研究技术路线

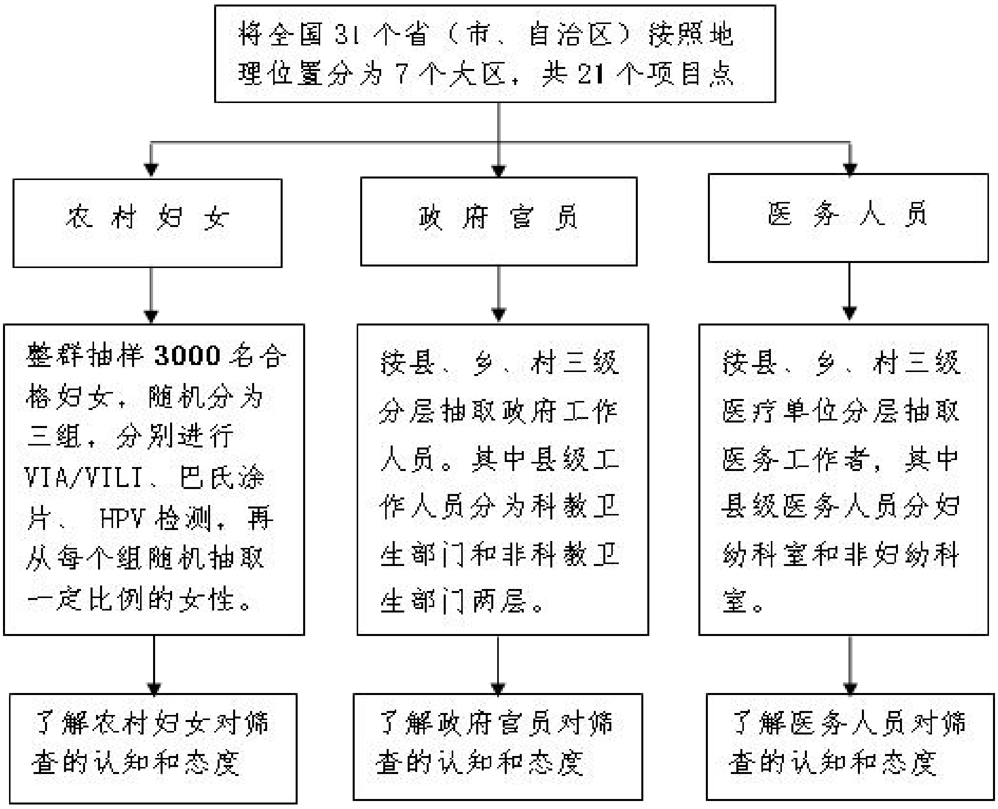

图3 认知及态度调查研究技术路线
