# Supplementary material for: Effectiveness of High-risk Human Papillomavirus Testing for Cervical Cancer Screening in China: A Multicenter, Open-label, Randomized Clinical Trial
Source: JAMA Oncol. 2020 Dec 30;7(2):1–9. doi: 10.1001/jamaoncol.2020.6575 (PMC7774051; doi:10.1001/jamaoncol.2020.6575)
Supplement: Supplement 2. — eFigure 1. National Map Presenting the Locations of the Study Sites eFigure 2. Study Profile eTable 1. Baseline Characteristics and Primary Screening Results for 24-month Followed-up and Lost Follow-up Participants eTable 2. Population and Outcomes for hrHPV Testing, Cytology, or VIA/VILI Arms at 24-month Follow-up Screening and the 24-month Cumulative Results eTable 3. Estimation of Triaging HPV-positive Women with Cytology, VIA/VILI, or Direct Colposcopy eTable 4. Outcomes for hrHPV Testing Arm, Cytology Arm, and VIA/VILI Arm at the 24-month Screening [file jamaoncol-e206575-s002.pdf]

Supplemental Online Content

J Zhang, Y Zhao, Y Dai, et al. Effectiveness of high-risk human papillomavirus testing for cervical cancer screening in China: a multicenter, open-label, randomized clinical trial. *JAMA Oncol*. Published online December 30, 2020. doi:10.1001/jamaoncol.2020.6575.

eFigure 1. National Map Presenting the Locations of the Study Sites

eFigure 2. Study Profile

eTable 1. Baseline Characteristics and Primary Screening Results for 24-month Followed-up and Lost Follow-up Participants

eTable 2. Population and Outcomes for hrHPV Testing, Cytology, or VIA/VILI Arms at 24-month Follow-up Screening and the 24-month Cumulative Results

eTable 3. Estimation of Triaging HPV-positive Women with Cytology, VIA/VILI, or Direct Colposcopy

eTable 4. Outcomes for hrHPV Testing Arm, Cytology Arm, and VIA/VILI Arm at the 24-month Screening

This supplemental material has been provided by the authors to give readers additional information about their work.

© 2020 Zhang et al. *JAMA Oncology.*

eFigure 1. National Map Presenting the Locations of the Study Sites

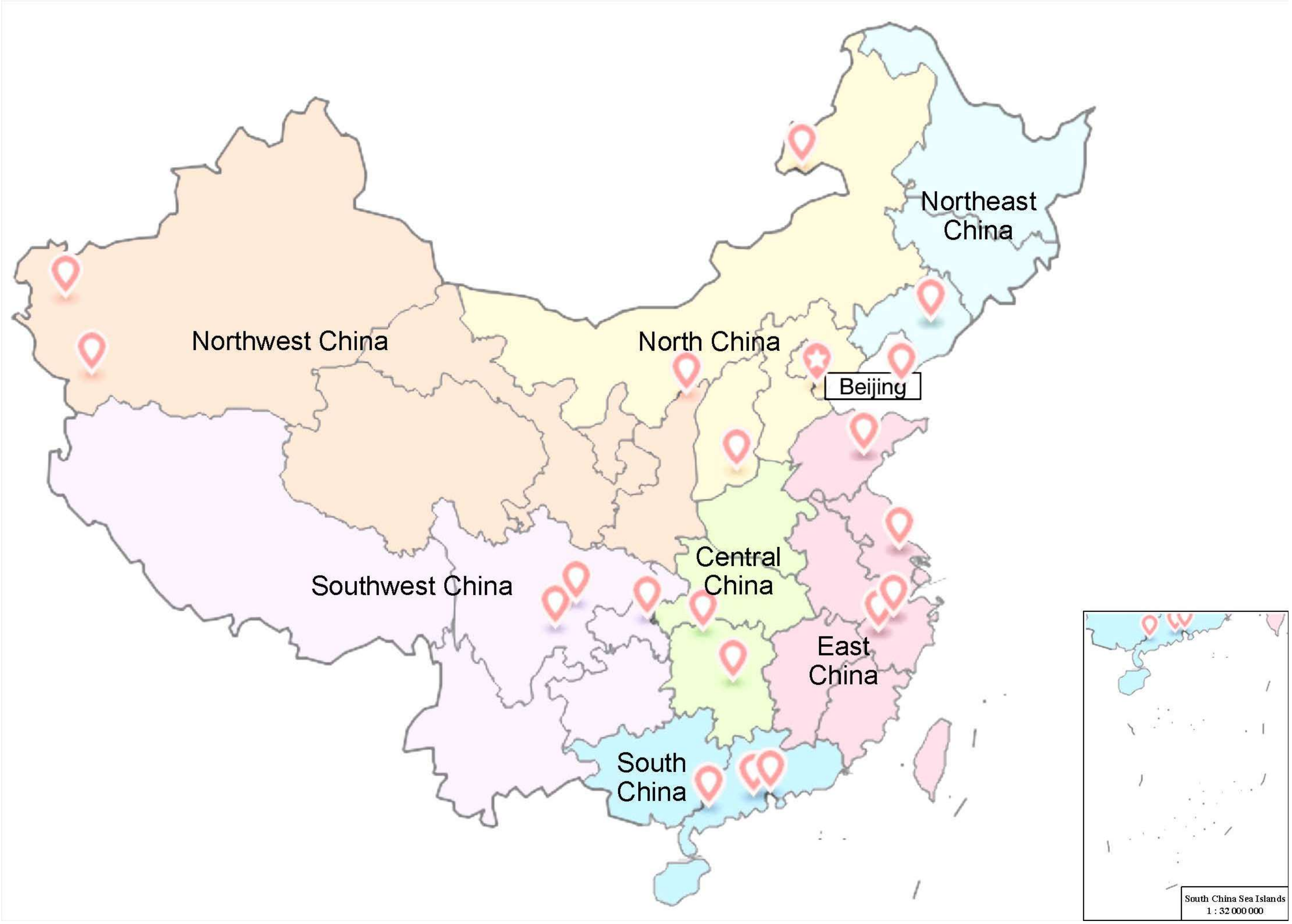

© 2020 Zhang et al. *JAMA Oncology.*

eFigure 2a. Study Profile for Urban Sites

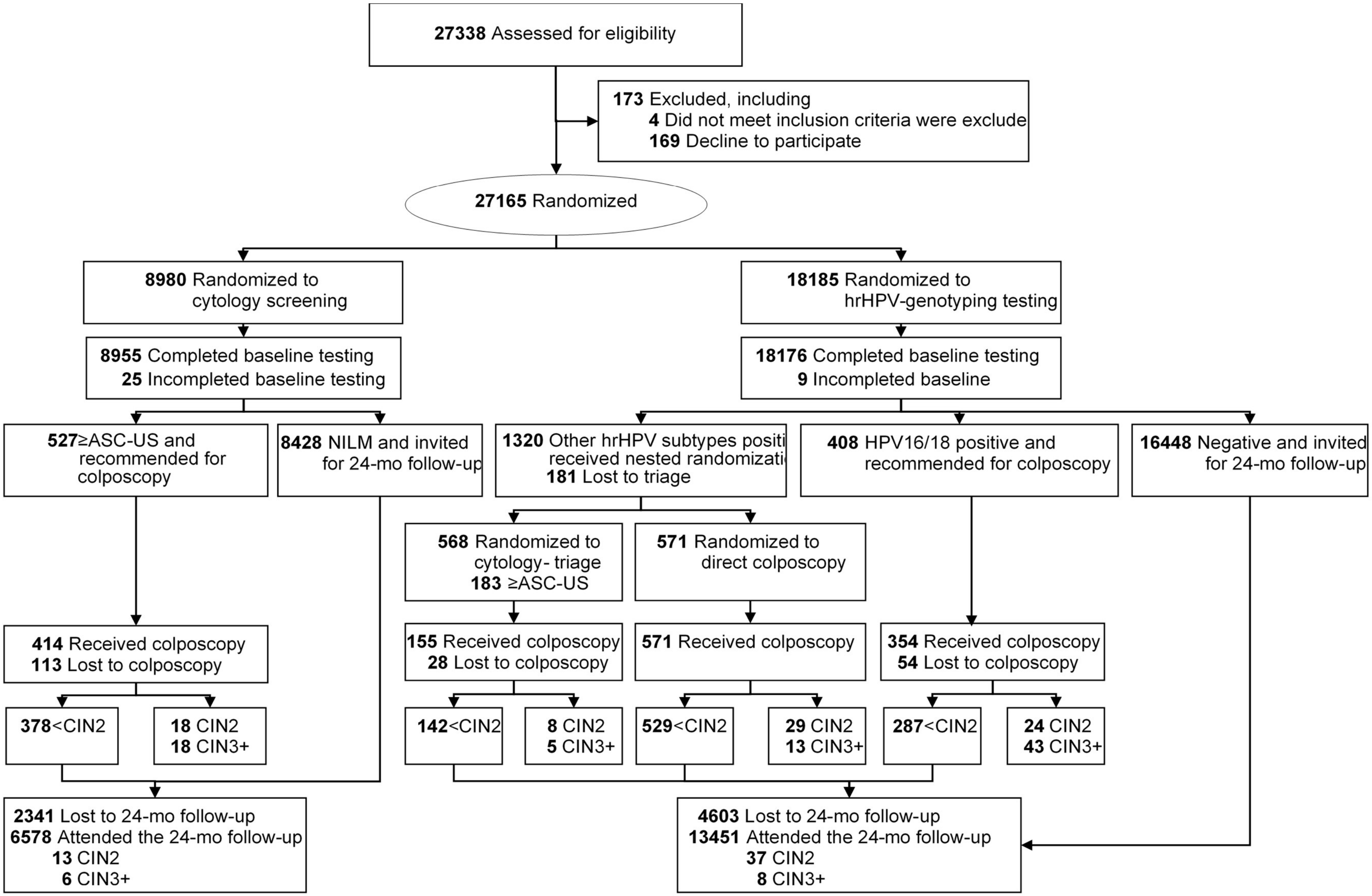

© 2020 Zhang et al. *JAMA Oncology.*

* HrHPV, high-risk human papillomavirus; ASC-US, atypical squamous cells of undetermined significance; NILM, negative for intraepithelial lesion or malignancy; CIN, cervical intraepithelial neoplasia.

eFigure 2b. Study Profile for Rural Sites

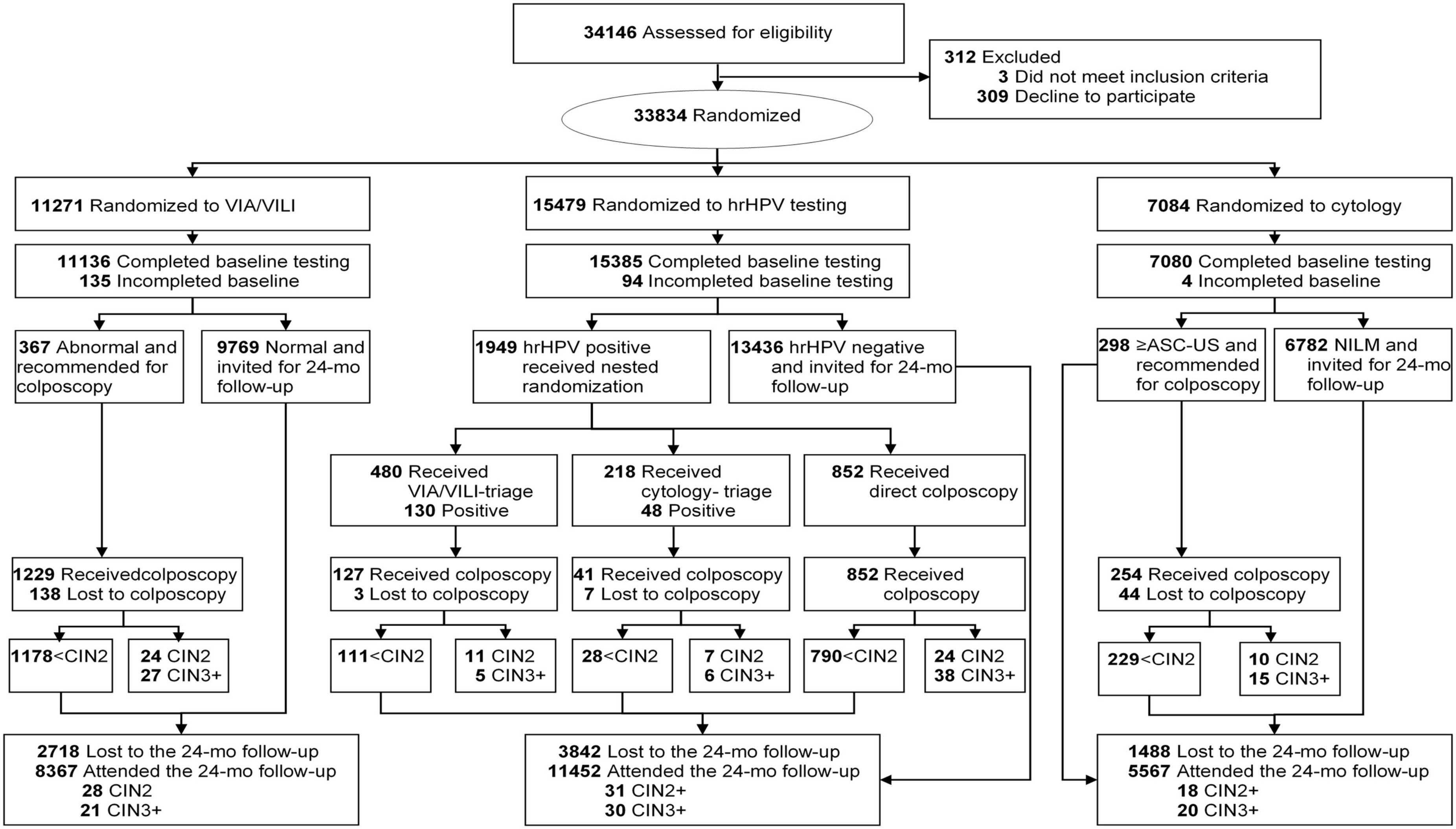

* VIA/VILI, Visual inspection with acetic acid and Lugol`s iodine; hrHPV, high-risk human papillomavirus; ASC-US, atypical squamous cells of undetermined significance; NILM, negative for intraepithelial lesion or malignancy; CIN, cervical intraepithelial neoplasia.

© 2020 Zhang et al. *JAMA Oncology.*

eTable 1. Baseline Characteristics and Primary Screening Results for 24-month Followed-up and Lost Follow-up Participants

	hrHPV arm		Cytology arm		VIA/VILI arm	
	Followed-up (n=24903) No. (%)	Lost Follow-up (n=8445) No. (%)	Followed-up (n=12145) No. (%)	Lost Follow-up (n=3829) No. (%)	Followed-up (n=8367) No. (%)	Lost Follow-up (n=2718) No. (%)
**Age**						
**Median (IQR)**	46.8 (41.0-52.0)	46.6 (41.0-52.0)	47.3 (42.0-52.0)	47.2 (41.0-52.0)	47.8 (42.0-53.0)	47.0 (40.0-53.0)
35-44	10159 (40.8)	3744 (44.3)	4688 (38.6)	1549 (40.5)	3086 (36.9)	1140 (41.9)
45-54	10905 (43.8)	3255 (38.5)	5340 (44.0)	1533 (40.0)	3534 (42.2)	1019 (37.5)
55-64	3839 (15.4)	1446 (17.1)	2117 (17.4)	747 (19.5)	1747 (20.9)	559 (20.6)
**Marriage**						
Single	59 (0.2)	22 (0.3)	36 (0.3)	14 (0.4)	10 (0.1)	1 (0.04)
Married	23478 (95.2)	7924 (94.6)	11578 (95.4)	3612 (94.4)	8005 (96.2)	2575 (95.3)
Divorced	530 (2.2)	229 (2.7)	254 (2.1)	109 (2.9)	88 (1.1)	47 (1.7)
Widowed	579 (2.4)	197 (2.4)	247 (2.0)	88 (2.3)	205 (2.5)	72 (2.7)
Others	12 (0.1)	6 (0.1)	19 (0.2)	4 (0.1)	15 (0.2)	6 (0.2)
**Annual family income (RMB/Year)**						
<30000	10593 (43.1)	3866 (46.2)	5487 (45.4)	1730 (45.3)	4500 (54.1)	1464 (54.2)
30000-60000	9067 (36.9)	2952 (35.3)	4261 (35.2)	1248 (32.7)	2999 (36.1)	988 (36.6)
60000-100000	3104 (12.6)	955 (11.4)	1470 (12.2)	517 (13.5)	535 (6.4)	174 (6.5)
>100000	1844 (7.5)	588 (7.0)	879 (7.3)	325 (8.5)	280 (3.4)	73 (2.7)
**Smoke**						
Current or former smoker	511 (2.1)	200 (2.4)	363 (3.0)	100 (2.6)	244 (3.0)	84 (3.1)
Never or rarely	23950 (97.9)	8126 (97.6)	11679 (97.0)	3709 (97.4)	8038 (97.1)	2609 (96.9)
**Menopause**						
No	14638 (60.5)	5223 (63.2)	7191 (59.9)	2302 (60.6)	4577 (56.0)	1591 (59.7)
Perimenopause	1654 (6.8)	472 (5.7)	818 (6.8)	201 (5.3)	483 (5.9)	140 (5.3)
Postmenopausal	7901 (32.7)	2567 (31.1)	3994 (33.3)	1294 (34.1)	3113 (38.1)	933 (35.0)
**Sexual partners in recent 3 years**						
None	1451 (5.8)	434 (5.1)	425 (3.5)	153 (4.0)	416 (5.0)	141 (5.2)
1	23280 (93.5)	7936 (94.0)	11636 (95.9)	3646 (95.3)	7891 (94.4)	2566 (94.4)
>=2	171 (0.7)	72 (0.9)	73 (0.6)	27 (0.7)	52 (0.6)	10 (0.4)
**Baseline screening results**						
Positive	2551 (10.2)	913 (10.8)	571 (4.7)	193 (5.0)	998 (11.9)	318 (11.7)

© 2020 Zhang et al. *JAMA Oncology.*

eTable 2. Population and Outcomes for hrHPV Testing, Cytology, or VIA/VILI Arms at 24-month Follow-up Screening and the 24-month Cumulative Results.

	Urban Sites			Rural Sites				
	hrHPV testing arm[a] (n=18176) No. (%) [95%CI]	Cytology arm (n=8955) No. (%) [95%CI]	hrHPV vs. Cytology RR (95%CI)	hrHPV testing arm[a] (n=15385) No. (%) [95%CI]	Cytology arm (n=7080) No. (%) [95%CI]	VIA/VILI arm (n=11136) No. (%) [95%CI]	hrHPV testing vs. Cytology RR (95%CI)	hrHPV testing vs. VIA/VILI RR (95%CI)
**24-mo Follow-up Screening[b]**								
Participants	13451 (74.0) [73.4-74.6]	6578 (73.5) [72.5-74.4]		11452 (74.4) [73.7-75.1]	5567 (78.6) [77.7-79.6]	8367 (75.1) [74.3-75.9]		
Positive	1782 (9.8) [9.4-10.2]	807 (9.0) [8.4-9.6]	1.1 (1.01-1.2)	1832 (11.9) [11.4-12.4]	1056 (14.9) [14.1-15.8]	1372 (12.3) [11.7-12.9]	0.8 (0.7-0.9)	1.0 (0.9-1.03)
Colposcopy	1470 (8.1) [7.7-8.5]	665 (7.4) [6.9-8.0]	1.1 (0.99-1.2)	1435 (9.3) [8.9-9.8]	970 (13.7) [12.9-14.5]	1148 (10.3) [9.8-10.9]	0.7 (0.6-0.7)	0.9 (0.8-0.97)
CIN2+ yield	45 (0.2) [0.2-0.3]	19 (0.2) [0.1-0.3]	1.2 (0.7-2.0)	61 (0.4) [0.3-0.5]	38 (0.5) [0.4-0.7]	49 (0.4) [0.3-0.6]	0.8 (0.5-1.1)	0.9 (0.6-1.3)
CIN3+ yield[c]	8 (0.04) [0.02-0.1]	6 (0.1) [0.03-0.2]	0.7 (0.2-1.9)	30 (0.2) [0.1-0.3]	20 (0.3) [0.2-0.4]	21 (0.2) [0.1-0.3]	0.7 (0.4-1.2)	1.0 (0.6-1.8)
**24-mo Cumulative Results**								
Positive	3510 (19.3) [18.7-19.9]	1334 (14.9) [14.2-15.7]	1.3 (1.2-1.4)	3781 (24.6) [23.9-25.3]	1354 (19.1) [18.2-20.1]	2739 (24.6) [23.8-25.4]	1.3 (1.2-1.4)	1.0 (0.9-1.04)
Colposcopy	2550 (14.0) [13.5-14.5]	1079 (12.0) [11.4-12.7]	1.2 (1.1-1.2)	2455 (16.0) [15.4-16.5]	1224 (17.3) [16.4-18.2]	2377 (21.3) [20.6-22.1]	0.9 (0.9-0.99)	0.8 (0.7-0.8)
CIN2+ yield	167 (0.9) [0.8-1.1]	55 (0.6) [0.5-0.8]	1.5 (1.1-2.0)	152 (1.0) [0.8-1.2]	63 (0.9) [0.7-1.1]	100 (0.9) [0.7-1.1]	1.1 (0.8-1.5)	1.1 (0.9-1.4）
CIN3+ yield[c]	69 (0.4) [0.3-0.5]	24 (0.3) [0.2-0.4]	1.4 (0.9-2.3)	79 (0.5) [0.4-0.6]	35 (0.5) [0.4-0.7]	48 (0.4) [0.3-0.6]	1.0 (0.7-1.5)	1.2 (0.8-1.8)

HrHPV=high-risk human papillomavirus. CIN=cervical intraepithelial neoplasia. VIA/VILI=visual inspection with acetic acid and Lugol's iodine.
a. hrHPV arm=All women have had hrHPV testing as primary screening at baseline, regardless of the triage tests.
b.At the 24-mo screening, women in urban sites were co-tested by hrHPV testing and cytology, women in rural sites were screened by hrHPV testing, cytology and VIA/VILI combined. Any positive result was referred to colposcopy.
c. At baseline screening, 10 cervical cancers were detected in urban sites, and 6 cervical cancers were detected in rural sites; at the 24-mo follow-up screening, 4 cervical cancers were detected in urban sites, and 8 cervical cancers were detected in rural sites.

© 2020 Zhang et al. *JAMA Oncology.*

eTable 3. Estimation of Triaging HPV-positive Women with Cytology, VIA/VILI, or Direct Colposcopy

	Urban Sites		Rural Sites		
	Direct COLP[a] No. (%) [95%CI]	Cytology-Triage[b] No. (%) [95%CI]	Direct COLP[a] No. (%) [95%CI]	Cytology-Triage[b] No. (%) [95%CI]	VIA/VILI-Triage No. (%) [95%CI]
**Baseline Screening**					
Positive	1728.0 (9.5) [9.1-9.9]	833.3 (4.6) [4.3-4.9]	1949.0 (12.7) [12.2-13.2]	429.1 (2.8) [2.3-3.2]	527.9 (3.4) [3.0-3.8]
Colposcopy	1674.0 (9.2) [8.8-9.6]	714.2 (3.9) [3.7-4.2]	1949.0 (12.7) [12.2-13.2]	366.6 (2.4) [2.0-2.9]	515.7 (3.4) [2.9-3.7]
CIN2+ yield	164.1 (0.9) [0.8-1.1]	97.2 (0.5) [0.4-0.7]	141.8 (0.9) [0.8-1.2]	116.2 (0.8) [0.5-1.05]	65.0 (0.4) [0.3-0.6]
CIN3+ yield	73.1 (0.4) [0.3-0.5]	54.6 (0.3) [0.2-0.4]	86.9 (0.6) [0.4-0.8]	53.6 (0.3) [0.2-0.5]	20.3 (0.1) [0.04-0.2]
**24-mo Follow-up Screening**					
Positive	443.4 (2.4) [2.2-2.7]	482.5 (2.7) [2.4-2.9]	521.6 (3.4) [3.1-3.7]	670.5 (4.4) [4.1-4.7]	523.8 (3.4) [3.1-3.7]
Colposcopy	370.3 (2.0) [1.8-3.0]	399.7 (2.2) [2.0-2.4]	375.2 (2.4) [2.2-2.7]	616.9 (4.0) [3.7-4.3]	438.5 (2.9) [2.6-3.1]
CIN2+ yield	19.9 (0.1) [0.1-0.2]	24.6 (0.1) [0.1-0.2]	50.3 (0.3) [0.3-0.4]	62.6 (0.4) [0.3-0.5]	24.4 (0.2) [0.1-0.2]
CIN3+ yield	2.0 (0.01) [0.00-0.04]	4.3 (0.02) [0.01-0.1]	27.5 (0.2) [0.1-0.3]	8.9 (0.1) [0.03-0.1]	4.1 (0.03) [0.01-0.1]
**24-mo Cumulative Results**					
Positive	2171.4 (11.9) [11.5-12.4]	1315.7 (7.2) [6.9-7.6]	2470.6 (16.1) [15.5-16.7]	1099.7 (7.1) [6.8-7.6]	1051.6 (6.8) [6.5-7.2]
Colposcopy	2044.3 (11.2) [10.8-11.7]	1113.9 (6.1) [5.8-6.5]	2324.2 (15.1) [14.6-15.7]	983.4 (6.4) [6.0-6.8]	954.2 (6.2) [5.8-6.6]
CIN2+ yield	184.0 (1.0) [0.9-1.2]	121.8 (0.7) [0.6-0.8]	192.2 (1.3) [1.1-1.4]	178.8 (1.2) [1.0-1.3]	89.3 (0.7) [0.5-0.9]
CIN3+ yield	75.1 (0.4) [0.3-0.5]	58.9 (0.3) [0.3-0.4]	114.4 (0.7) [0.6-0.9]	62.6 (0.4) [0.3-0.5]	24.4 (0.2) [0.1-0.2]

All datas are adjusted for verification bias, not actual numbers. HrHPV=high-risk human papillomavirus. CIN=cervical intraepithelial neoplasia. VIA/VILI=visual inspection with acetic acid and Lugol's iodine.

a. Direct COLP=All hrHPV-positive women were referred to colposcopy directly.

b.Cytology-Triage=Women in urban sites with HPV16/18 positive referred to direct colposcopy, other hrHPV subtypes-positive had cytology-triage, cytology ASC-US+ referred to colposcopy; women in rural sites with positive hrHPV had cytology-triage, cytology ASC-US+ referred to colposcopy.

© 2020 Zhang et al. *JAMA Oncology.*

eTable 4:

Assuming all participants that missed follow up screening had a similar chance of having a positive follow-up screening results (that should called them back for colposcopy) as well as of negative (that colposcopy is not required), we imputed the outcome based on the observed data (Age at recruitment and baseline screen results).

Multiple imputation based on logistic regression was used to account for missing outcomes for women lost to follow-up. For imputation, enrollment follow-up screening results were dichotomized to be either positive (hrHPV or cytology [⩾ASCUS] or VIA/VILI [only for rural sites] positive) or all negative (hrHPV and cytology and VIA/VILI [only for rural sites] negative).

eTable 4 below shows the multiple imputation outcomes for hrHPV arm, cytology arm and VIA/VILI arm with positive and negative baseline results.

© 2020 Zhang et al. *JAMA Oncology.*

eTable 4. Imputed Outcomes for hrHPV Testing Arm, Cytology Arm, and VIA/VILI Arm at the 24-month Screening.

	Urban sites			Rural sites				
	**hrHPV testing arm (n=18176) %**	**Cytology arm (n=8955) %**	**hrHPV vs. Cytology RR (95%CI)**	**hrHPV testing arm (n=15385) %**	**Cytology arm (n=7080) %**	**VIA/VILI arm (n=11136) %**	**hrHPV testing vs. Cytology RR (95%CI)**	**hrHPV testing vs. VIA/VILI RR (95%CI)**
**24-mo Follow-up Screening [a]**								
Positive	13.5	12.4	1.1 (1.02-1.2)	16.5	18.1	16.6	0.9 (0.9-0.97)	1.0 (0.9-1.04)
CIN2+ yield	0.4	0.3	1.3 (0.9-2.0)	0.9	1.0	0.9	0.9 (0.7-1.2)	1.0 (0.9-1.04)
CIN3+ yield	0.1	0.1	0.8 (0.3-2.1)	0.4	0.5	0.3	0.8 (0.5-1.2)	1.2 (0.8-1.8)
**24-mo Cumulative Results**								
Positive	23.0	18.2	1.3 (1.2-1.3)	29.1	22.3	28.9	1.3 (1.2-1.4)	1.0 (0.97-1.04)
CIN2+ yield	1.1	0.7	1.5 (1.1-2.0)	1.5	1.3	1.4	1.1 (0.9-1.4)	1.1 (0.9-1.7)
CIN3+ yield	0.4	0.3	1.4 (0.9-2.3)	0.7	0.7	0.6	1.0 (0.7-1.5)	1.2 (0.8-1.8)
**24-mo Follow-up Screening (Primary hrHPV testing, cytology, or VIA/VILI) for Baseline Negative Women [a]**								
Positive	10.9	11.5	0.9 (0.9-1.01)	13.4	17.3	15.6	0.8 (0.7-0.8)	0.9 (0.8-0.9)
CIN2+ yield	0.3	0.3	1.1 (0.7-1.7)	0.5	0.8	0.8	0.7 (0.5-0.9)	0.7 (0.5-0.9)
CIN3+ yield	0.04	0.1	0.6 (0.2-1.8)	0.2	0.5	0.3	0.5 (0.3-0.8)	0.8 (0.5-1.2)

HrHPV=high-risk human papillomavirus. CIN=cervical intraepithelial neoplasia. VIA/VILI=visual inspection with acetic acid and Lugol's iodine.
a. At the 24-mo screening, women in urban sites were co-tested by hrHPV testing and cytology, women in rural sites were screened by hrHPV testing, cytology and VIA/VILI combined. Positive results included hrHPV positive, cytology ≥ASCUS or VIA/VILI-positive (only for rural sites).

© 2020 Zhang et al. *JAMA Oncology.*
