# Supplementary material for: Effectiveness of High-risk Human Papillomavirus Testing for Cervical Cancer Screening in China: A Multicenter, Open-label, Randomized Clinical Trial
Source: JAMA Oncol. 2020 Dec 30;7(2):1–9. doi: 10.1001/jamaoncol.2020.6575 (PMC7774051; doi:10.1001/jamaoncol.2020.6575)
Supplement: Supplement 3. — Data-Sharing Statement [file jamaoncol-e206575-s003.pdf]

Data Sharing Statement

Zhang. Effectiveness of High-risk Human Papillomavirus Testing for Cervical Cancer Screening in China. *JAMA Oncol*. Published December 30, 2020. doi:10.1001/jamaoncol.2020.6575

Data
Data available: Yes
Data types: Deidentified participant data, Data dictionary
How to access data: http://www.chictr.org.cn. Anyone request for data must be sent to qiaoy@cicams.ac.cn and langjh@hotmail.com
When available: With publication

Supporting Documents
Document types: None

Additional Information
Who can access the data: Researchers whose proposed use of the data has been approved that the data will be made available.
Types of analyses: The data will be made available for Meta-analysis and pooled-analysis which is for meaningful quantitative syntheses of research on the effects of public health interventions, as well as future directions for cancer guideline.
Mechanisms of data availability: The data will be made available if fulfilled the following criterias: A.With investigator or organization support. B. The proposal of data use has been approved by the corresponding authors. C. With a signed data access agreement.
